# Linking Transition Metal Concentration and Oxidative Potential in PM_2.5_ by Ascorbic Acid Assay via Quasi-Michaelis–Menten Mechanism

**DOI:** 10.1021/acs.est.5c09374

**Published:** 2025-10-21

**Authors:** Yuhuang Cheng, Hanzhe Chen, Jian Zhen Yu

**Affiliations:** † Department of Chemistry, 58207 Hong Kong University of Science & Technology, Hong Kong 999077, China; ‡ Division of Environment and Sustainability, Hong Kong University of Science & Technology, Hong Kong 999077, China

**Keywords:** oxidative potential, transition metals, ascorbic acid assay, humic-like substances, reactive oxygen species (ROS)

## Abstract

Transition metals (TMs) in the ambient fine particulate matter (PM_2.5_) catalyze the formation of multiple reactive oxidative species (ROS), such as superoxide, hydrogen peroxide, and hydroxyl radicals, in lung lining fluid, contributing to the oxidative potential (OP) of inhaled particles. Complexation with ambient organics, particularly humic-like substances (HULIS), further modulates TM-catalyzed ROS generation by influencing electron transfer processes. While OP measurements are widely reported for both ambient and laboratory samples, the detailed catalytic mechanisms and chemical kinetics underlying TM-induced OP remain under-investigated and poorly understood. Here, we systematically investigated the OP of Fe and Cu using ascorbic acid (AA) assay under varying conditions. Reaction kinetics and studies of OP dependence on TM concentration have led us to propose a quasi-Michaelis–Menten mechanistic framework that involves a TM–AA complex as a key intermediate for OP generation in the AA assay. This mechanism explains the observed nonlinear kinetics and dose–response behavior of AA depletion and hydroxyl radical production. We also explored the mixing effects between TMs and HULIS in generating OP. These findings clarify the mechanistic link between TM concentration and OP in the AA assay and provide a foundation for OP modeling based on PM_2.5_ chemical composition.

## Graphical abstract

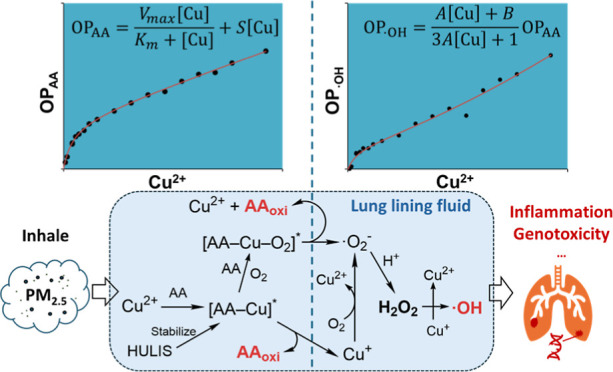

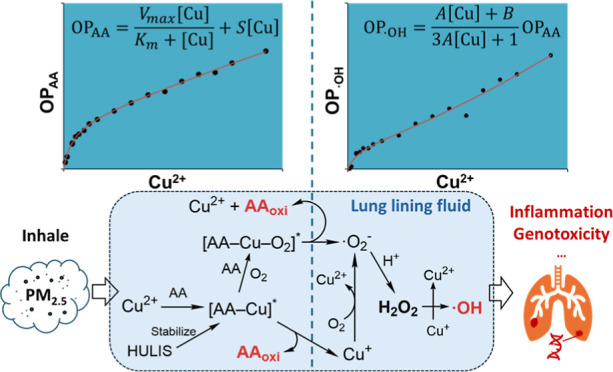

## 1 Introduction

Inhalation of ambient particulate matter with an aerodynamic diameter less than 2.5 μm (PM_2.5_) allows these particles to deposit in lung tissue or penetrate deep into the gas-exchange regions,^1,2^ resulting in adverse health effects and increased risk of mortality through multiple biochemical mechanisms.^3^ One key pathway involves the depletion of antioxidants, such as ascorbic acid (AA) and glutathione (GSH), by inhaled PM_2.5_, which leads to the generation of reactive oxygen species (ROS) and elevated oxidative stress in the body.^4–7^ To assess the oxidative potential (OP) of PM, several acellular assays have been developed. Among them, the AA assay measures the consumption of AA, a major antioxidant in lung lining fluid;^8^ the GSH assay quantifies the loss of GSH, an abundant cellular tripeptide with ROS-reducing capabilities;^9,10^ and the dithiothreitol (DTT) assay evaluates OP by simulating the consumption of cellular antioxidants such as GSH and NADPH.^11^

Transition metal (TM) ions, especially copper (Cu), iron (Fe), and manganese (Mn), are recognized as primary contributors to OP of PM_2.5_ due to their high redox activity and ability to facilitate electron transfer during ROS generation. Numerous studies using both laboratory-prepared metal solutions and water extracts of ambient PM have documented the significant role of TMs in OP, as measured by various assays.^12–16^ In addition to TMs, certain organic compounds can modulate ROS generation by forming complexes with metal ions.^7,17,18^ Among these, humic-like substances (HULIS) are particularly notable, as their abundant functional groups facilitate strong metal chelation and contribute to high OP activity in surrogate lung fluids.^19,20^

Previous studies have reported nonlinear relationship between TM concentration and DTT depletion rate^17,21,22^ and the synergistic effects between TMs and ambient organics.^23^ For the AA assay, some studies have presented empirical equations describing OP as a function of TM concentration.^12,24^ However, these relationships are typically regression-based and lack underlying chemical interpretation, limiting their broader applicability. While several investigations have explored the mechanisms of TM-induced AA consumption and ROS formation, they often characterize only isolated reactions or report step-specific rate constants without addressing overall OP generation.^25,26^ Other AA-focused studies provide kinetic parameters derived from empirical regression, without mechanistic chemistry foundations^13,15,22,27^ or propose multistep pathways that lack experimental validation of kinetics.^28–30^ As a result, there remains a need for a universal mechanism that quantitatively links TM concentration to OP in the AA assay. This gap complicates comparison of OP results across varying conditions and hinders a full understanding of the mixing effects between ambient organics and TM ions.

The aim of this study is to investigate the detailed kinetic processes and underlying chemical mechanisms of Cu-induced and Fe-induced OP in the AA assay,^18,31,32^ using both laboratory-prepared systems and ambient PM_2.5_ water extracts. We optimized and simplified the AA assay protocol by conducting reactions directly in 1.5 mL brown HPLC vials with integrated temperature control, which eliminates the need for solution transfer or additional quenching steps and improves procedural reproducibility. While our previous work^18^ primarily focused on elucidating the chemical interactions between transition metals and compounds containing specific functional groups (such as carboxylic acids and imidazoles), the present study shifts the emphasis to reaction kinetics and mechanistic modeling. Based on OP measurements under different reaction conditions, we propose a quasi-Michaelis–Menten mechanism to quantitatively describe OP as a function of metal concentration, with the metal–AA complex identified as a key reaction intermediate. Furthermore, we demonstrate that ambient HULIS can stabilize the metal–AA complex, thereby modulating electron transfer and resulting in a prolonged OP effect due to an extended lifetime of the reactive Cu^2+^ intermediate in ambient PM_2.5_ water extracts compared with lab-prepared single-metal solutions. These findings provide deeper chemical insight into TM-induced OP in the AA assay and offer a valuable framework for OP calculation and modeling based on TM concentrations in ambient PM.

## 2 Experiment Methods

### 2.1 Chemicals and Buffers

Copper­(II) perchlorate (98%), iron­(II) sulfate heptahydrate (>99%), potassium phosphate monobasic (KH_2_PO_4_), potassium phosphate dibasic (K_2_HPO_4_), and l-ascorbic acid (ACS grade) were obtained from Sigma-Aldrich. Benzoic acid (ACS grade) was purchased from AcrosOrganics, sodium chloride (99.5%) from AnalaR NORMAPUR, and Chelex 100 resin (100–200 mesh, sodium form) from Biorad. Phosphate buffer (PB, 77.5 mM K_2_HPO_4_ and 22.5 mM KH_2_PO_4_, pH 7.4) and phosphate-buffered saline with benzoate (PBSB; 10 mM benzoic acid, 114 mM NaCl, 7.8 mM K_2_HPO_4_, 2.2 mM KH_2_PO_4_, pH 7.4) were prepared and treated with Chelex 100 resin to remove trace metals, then stored in acid-washed (10% HNO_3_, 24 h) polypropylene bottles. Details on Chelex treatment and validation are provided in our previous study.^17^

### 2.2 Ambient Samples

Ambient PM_2.5_ samples used in this work were collected on the rooftop of the library at the China University of Petroleum located in Changping District, Beijing, China (40°13′7″N, 116°14′49″E). Sampling was conducted every 6 days from April 2, 2016, to April 27, 2017, with each collection spanning 24 h (midnight to midnight). A high-volume PM_2.5_ sampler (TH-1000 series, Tianhong Corp., Wuhan, China) equipped with prebaked quartz fiber filters (8 × 10 in.) operated at a flow rate of 1.13 m^3^/min. Field blank filters were collected under identical conditions. After sampling, filters were wrapped in prebaked aluminum foil, transported with ice packs, and stored at −18 °C until analysis.

Each filter portion (20 cm^2^) was sectioned from the parent filter, cut into small pieces, and extracted with 20 mL ultrapure water by sonication for 60 min. Extracts were filtered (0.45 μm PTFE) to remove particulates. For fractionation, 6 mL of extract was loaded onto an Oasis HLB solid-phase extraction cartridge (30 mm). The hydrophilic (HPI) fraction was collected as the eluent; the hydrophobic (HPO) fraction was eluted with 6 mL methanol, dried under high-purity N_2_, and reconstituted in 6 mL water to restore original concentration. All extracts were stored at 4 °C prior to analysis. Concentrations of Fe, Cu, and Mn were determined using inductively coupled plasma optical emission spectrometry (ICP-OES, PerkinElmer Avio 2000).

### 2.3 OP by AA Assay

The acellular AA assay procedure in this study is a simplified version of the methods established in previous studies.^12,18^ Briefly, 0.2 mL of sample was mixed with 0.78 mL PBSB and equilibrated at 37 °C for 30 min. The reaction was initiated by adding 200 μM AA (20 μL, 10 mM). Sodium benzoate (BA) in PBSB acted as a ^•^OH scavenger, yielding *p*-hydroxybenzoic acid (*p*-HBA) upon reaction with ^•^OH. AA and *p*-HBA concentrations were monitored every 16 min using an HPLC system (Waters e2695) with a photodiode array detector (Waters 2998). Unlike the method used in previous studies that keeps the reaction tube in an oven, temperature was controlled using the HPLC system’s integrated module, allowing reactions to proceed directly in 1.5 mL brown HPLC vials, eliminating the need for solution transfer or additional quenching steps. The HPLC gradient details are provided in Lin and Yu (2020).^18^

The accumulated ^•^OH concentration was calculated as

1 [OH·]=[p‐HBA]Yp‐HBA×fBA

where [*p-*HBA] is the concentration of *p*-HBA, *Y*_*p*‑HBA_ is the molar yield of *p*-HBA from BA (0.215 ± 0.018),^31^ and *f*_BA_ is the fraction of ^•^OH that reacts with BA, calculated as

2 fBA=kBA[BA][OH·]kBA[BA][OH·]+∑iki[Xi][OH·]

where [BA] and [X_*i*_] are concentrations of BA and other components that react with ^•^OH (e.g., AA, and other water-soluble organics from the ambient samples); *k*_BA_ and *k*_*i*_ are the rate constants, respectively. As [BA] is in large excess compared to any other organics in the system, *f*_BA_ is assumed to be 1. The percentage of AA consumed at reaction time *t* is calculated as

3 $${\Delta AA}_{t}\;\left(\%\right)=\frac{A_{0}-A}{A_{0}}\times 100\%$$

where *A*_0_ and *A* are the peak areas of AA at reaction time 0 and *t* min, respectively. OP_•OH_ and OP_AA_, i.e., the ^•^OH formation rate and AA loss rate in the system, respectively, are the regression slopes of [^•^OH] (μM) and ΔAA_*t*_ (% or μM) versus reaction time *t* (h), respectively.

## 3 Results and Discussion

### 3.1 Cu-Catalyzed AA Oxidation and ^•^OH Formation

#### 3.1.1 Mechanistic Insights into AA Oxidation at 37 °C

The cumulative loss of AA over reaction time exhibited distinct trends depending on copper concentration. High Cu samples showed a decreasing AA loss rate, while low Cu samples displayed a nearly linear trend (Figure 1a). This linearity indicates apparent zero-order kinetics with respect to AA (i.e., observed AA loss rate remaining constant and is independent of AA at a fixed Cu^2+^ level). To investigate the underlying reaction mechanism, we calculated the normalized AA loss per reaction interval (i.e., AA loss in each 16 min interval divided by the average AA concentration during that interval) and plotted the results over time (Figure 1b). If AA oxidation followed first-order reaction kinetics with respect to AA (i.e., rate = *k*[AA]), normalization would yield a flat trend in Figure 1b, as the dependency on AA concentration would be eliminated. However, high Cu samples showed a decreasing trend, indicating that the reaction does not follow first-order reaction kinetics. We then considered a second-order reaction model, where the reaction rate is proportional to both AA and Cu^2+^ (rate = *k*[AA]­[Cu^2+^]. In this scenario, the normalized AA loss reflects changes in Cu^2+^ concentration over time. The observed decrease in normalized AA loss suggests that Cu^2+^ is being consumed during the reaction.

**Figure 1:**
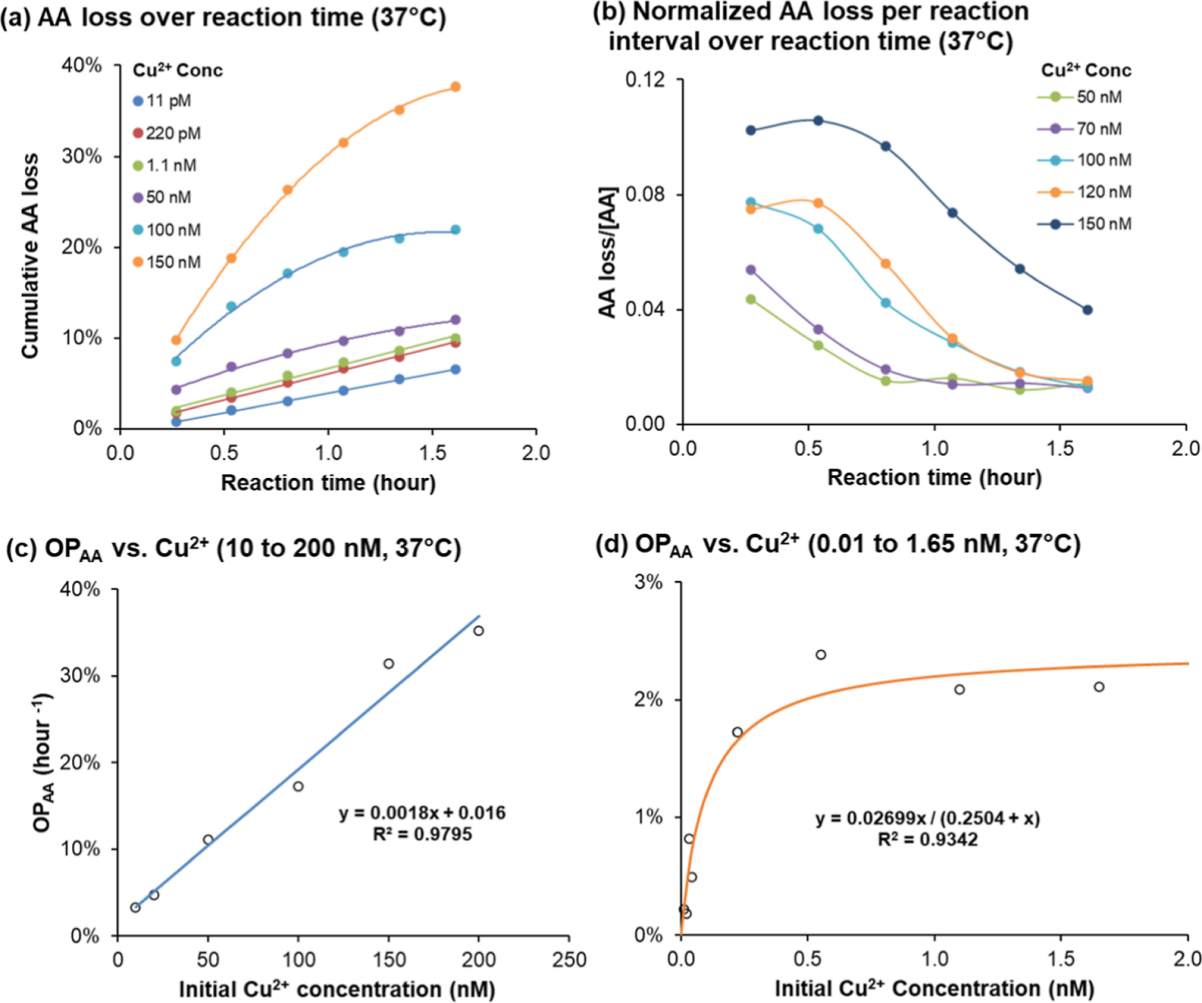
OP_AA_ reaction kinetics and dependence on Cu^2+^ concentration at 37 °C. (a) Cumulative percentage AA loss over reaction time up to 100 min at six different Cu^2+^ levels; (b) normalized AA loss per reaction interval versus reaction time; (c) percentage OP_AA_ (h^–1^) as a function of initial Cu^2+^ levels in the higher range of 10–200 μM; and (d) percentage OP_AA_ (h^–1^) as a function of initial Cu^2+^ levels in the lower range of 0.01–1.65 μM.

The above observation has led us to ask the question: what is the nonreactive form that Cu^2+^ turned into? Previous studies on Cu^2+^-induced AA oxidation have reported the formation of Cu^+^ as a reaction product.^27,33^ Although their experimental conditions differ from surrogate lung fluid, these findings suggest that the nonreactive form is likely Cu^+^. If AA had simply reduced Cu^2+^ to Cu^+^, the amount of AA oxidized would then closely match Cu^2+^ consumed. However, in our experiments, the maximum Cu^2+^ concentration was over 1000 times lower than the initial AA concentration, while cumulative AA loss after 1.6 h already reached up to 37%—far exceeding the initial Cu^2+^ present. This demonstrates that Cu^2+^ not only acts as a direct oxidant but also catalyzes AA oxidation, with catalysis dominating under our conditions.

In low Cu samples, the small cumulative AA loss allows AA concentration to be treated as nearly constant, and the reaction rate is sufficiently low that Cu^2+^ loss remains negligible. As a result, the second-order reaction approximates zero-order kinetics, consistent with the linear trend observed in Figure 1a.

To further test the hypothesized second-order kinetics, we examined the AA depletion rate as a function of Cu^2+^ concentration. For high Cu samples, where the AA loss rate decreases over time, OP_AA_ (expressed as the percentage AA loss rate) was calculated using only the first two time-points, ensuring both AA and Cu^2+^ concentrations remained near their initial values. For low Cu samples, which exhibit apparent zero-order kinetics, OP_AA_ was determined from the linear regression slope of cumulative AA loss versus reaction time. As shown in Figure 1c, OP_AA_ for high Cu samples increases linearly with Cu^2+^ concentration, consistent with second-order kinetics: because AA concentrations are relatively constant early in the reaction, the rate (*k*[AA]­[Cu^2+^]) is effectively proportional to the initial Cu^2+^ concentration. One expects that this relationship should also apply to low Cu samples, given the minimal change in AA concentration. However, the expanded view of low Cu samples in Figure 1d reveals a clear nonlinear relationship between OP_AA_ and Cu^2+^ concentration, indicating that second-order kinetics does not hold in this region. Instead, this nonlinear trend at low Cu concentrations closely fits a Michaelis–Menten-like model.

The classical Michaelis–Menten mechanism, illustrated in Scheme S1 in the Supporting Information, describes a substrate–enzyme system and yields zero-order kinetics at high substrate concentrations, which is similar to the apparent zero-order behavior observed for low Cu samples. However, the AA–Cu system in our acellular assay is more complex, and the classical Michaelis–Menten model does not fully capture the kinetics, particularly at high Cu concentrations. Our experimental data suggests that neither a simple second-order reaction nor the original Michaelis–Menten mechanism can universally describe AA oxidation across the entire Cu concentration range; each explains only a subset of the observed behaviors. Additionally, the Michaelis–Menten-like fit applies primarily to the low Cu region (Figure 1d), which sees small differences between sample points and is inherently associated with larger measurement uncertainty due to the small AA depletion. To establish a universal mechanism for Cu-induced AA oxidation, more data of higher measurement quality in the Michaelis–Menten-like region are required. We addressed this by conducting the OP_AA_ assay at a significantly lower temperature (15 °C) than 37 °C, as detailed in the following section.

#### 3.1.2 AA Oxidation at 15 °C

According to Arrhenius law, lowing the reaction temperature should slow the reaction rate for Cu^2+^-induced AA oxidation, potentially expanding the Cu^2+^ concentration range over which Michaelis–Menten-like kinetics (i.e., the transition from the linear to the plateau regime) are observable and thereby improving data quality. To test this, we conducted AA oxidation experiments at 15 °C with Cu^2+^ levels ranging from 10 to 1200 nM. As shown in Figure 2a, the cumulative AA loss over time is linear across all tested Cu^2+^ concentrations at 15 °C, allowing the apparent zero-order AA depletion rate, derived from linear regression, to be used as OP_AA_ for further analysis.

**Figure 2:**
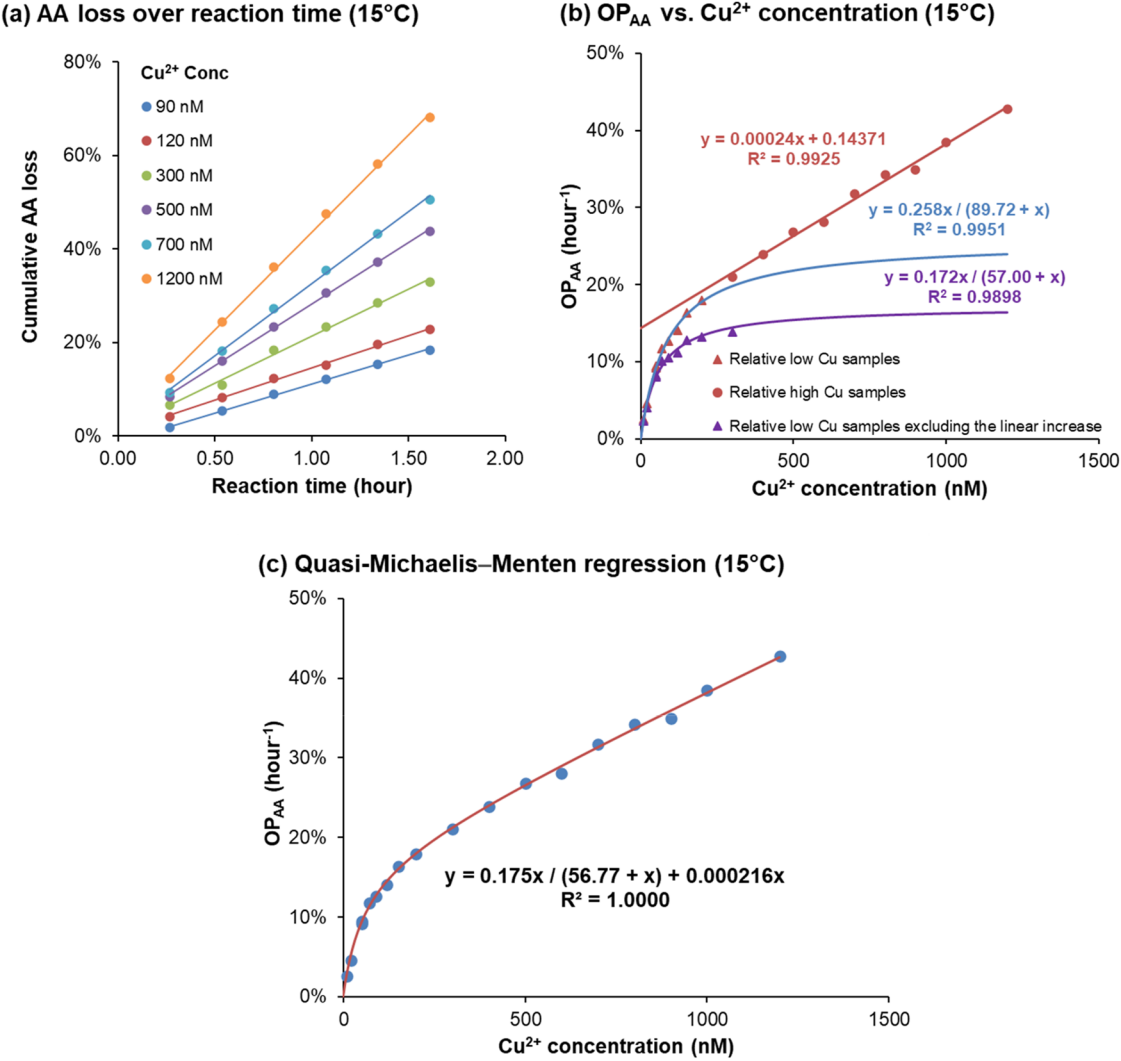
OP_AA_ reaction kinetics and dependence on Cu^2+^ concentration at 15 °C. (a) Cumulative percentage AA loss over time for different levels of Cu^2+^; (b) percentage OP_AA_ (h^–1^) curve over Cu^2+^ concentration at 15 °C: “red line with red dots” indicating linear regression for relatively high Cu samples, “blue line with red triangles” indicating Michaelis–Menten regression for relatively low Cu samples, and “purple line with purple triangles” indicating Michaelis–Menten regression for relatively low Cu samples after subtracting the linear increase in OP_AA_ based on the red line’s regression slope; and (c) direct quasi-Michaelis–Menten regression result based on eq 8.

Examining the relationship between OP_AA_ and Cu^2+^ concentration (Figure 2b), we observe a trend similar to that at 37 °C (Figure 1c,d), that is, the response splits at approximately 200 nM Cu^2+^ into a Michaelis–Menten-like region at low concentrations and a linear region at higher concentrations. However, unlike at 37 °C, the linear increase of OP_AA_ with Cu^2+^ at higher concentrations still reflects apparent zero-order kinetics at 15 °C. Here, the second-order kinetic model previously proposed for high Cu^2+^ at 37 °C is no longer applicable. For example, with an initial Cu^2+^ concentration of 1200 nM, the cumulative AA loss reaches 70% in 1.6 h (the orange line in Figure 2a), which will give a nonconstant *k*[AA]­[Cu^2+^] value even if the Cu^2+^ loss is negligible during the reaction.

#### 3.1.3 The Quasi-Michaelis–Menten Mechanism

The kinetics data shown in the preceding two subsections indicates that the AA loss rate depends on catalytic Cu^2+^ rather than on substrate AA concentration. This rate dependence has led us to suggest that the Cu^2+^–AA complex is the equivalent reactant. Given that AA is present in large excess over Cu^2+^, Cu^2+^ ions would rapidly form the Cu^2+^–AA complex, making its concentration effectively determined by Cu^2+^ levels in the system. This creates an equivalent binary system (Cu^2+^–AA complex and O_2_), consistent with the Michaelis–Menten-like kinetics observed at low Cu^2+^ concentrations. Based on this, we propose a quasi-Michaelis–Menten mechanism, illustrated in Scheme 1, to explain Cu^2+^-induced AA oxidation in the AA assay.

**Scheme 1:**
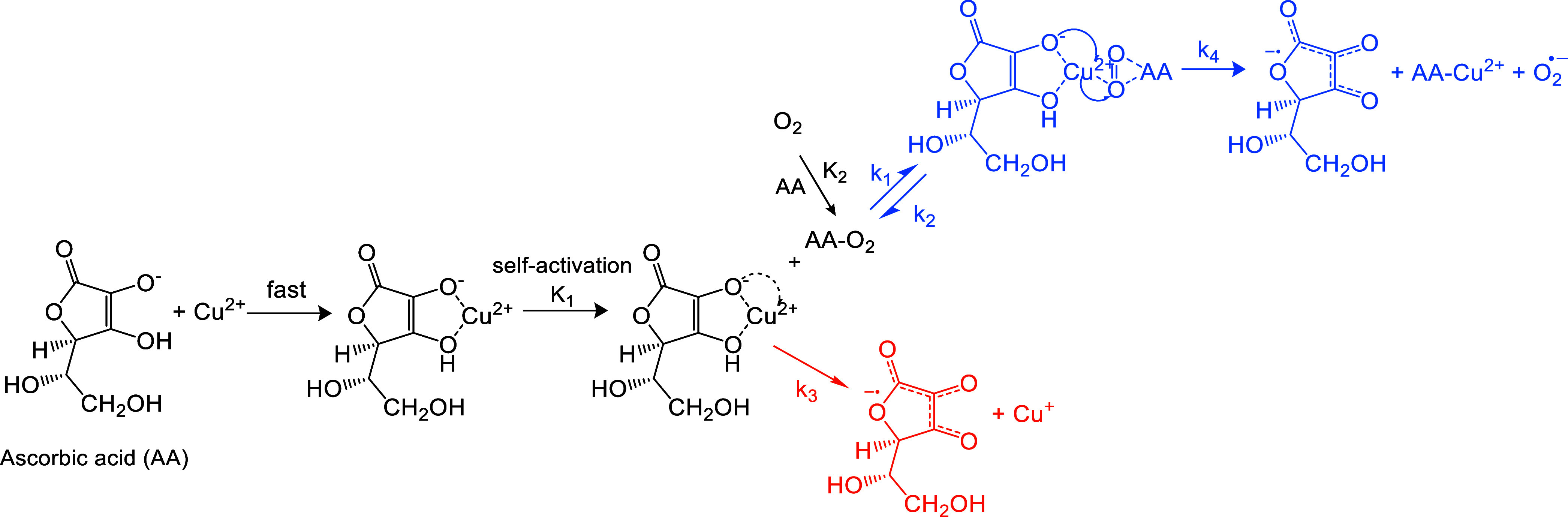
Quasi-Michaelis–Menten Mechanism for Cu^2+^-Induced AA Oxidation

The AA oxidation process begins with the rapid formation of a Cu^2+^–AA complex, followed by a self-activation step that produces the intermediate Cu^(2−δ)+^–AA^δ+^. This self-activation step is necessary for understanding subsequent Cu^+^ chemistry. Previous research on Cu^2+^-catalyzed AA oxidation reported a brown-colored intermediate prior to the formation of Cu­(I), which was attributed to a Cu^2+^–AA complex, with a corresponding formation constant provided.^33^ However, we propose that the intermediate they observed is not the original Cu^2+^–AA complex, but instead the intermediate Cu^(2−δ)+^–AA^δ+^, as its brown color differs from the typical blue color of other Cu^2+^ complexes (e.g., [Cu­(NH_3_)_4_(H_2_O)_2_]^2+^ and hemocyanin). Using their formation constant, we estimate that only about 1% of initial Cu^2+^ would exist as this intermediate in our system. In contrast, according to another study by Mahata et al.,^34^ all Cu^2+^ would chelate with AA when the Cu^2+^/AA ratio is 1/4.^34^ Given our much lower Cu^2+^/AA ratio (<1/100), almost all Cu^2+^ should rapidly form the Cu^2+^–AA complex. Thus, the intermediate in such a low level could not be the original Cu^2+^–AA complex but is more likely Cu^(2−δ)+^–AA^δ+^ instead. Based on the assumptions above, we can express the relationship between Cu^2+^–AA and Cu^(2−δ)+^–AA^δ+^ with the formation constant *K*_1_ as eq S10.

AA can also be directly oxidized by dissolved oxygen in the absence of metal ions in aqueous systems. One previous kinetic study indicates that this reaction is first order with respect to oxygen, but independent of AA concentration.^35^ If AA–O_2_ is considered an intermediate, its concentration should be proportional to dissolved oxygen, defined by a constant *K*_2_, as expressed in eq S11.

The intermediate Cu^(2−δ)+^–AA^δ+^ can undergo two reaction pathways: direct dissociation to form Cu^+^ and oxidized AA, or reaction with AA–O_2_, leading to a Michaelis–Menten-like process. For the latter, analogous to the substrate–enzyme intermediate in classical Michaelis–Menten kinetics (Scheme S1 and Text S1), we hypothesize the formation of a three-body intermediate, AA–O_2_–Cu^(2−δ)+^–AA^δ+^. A similar three-body intermediate has been proposed in a previous study of TM-catalyzed AA oxidation by Khan and Martell in 1967,^28^ though the authors did not observe the quasi-Michaelis–Menten behavior, likely due to the use of much higher Cu and AA concentrations in their study. Ultimately, the intermediate dissociates to produce ^•^O_2_^–^, Cu^2+^, and oxidized AA. The regenerated Cu^2+^ rapidly complexes with AA, completing the catalytic cycle. Applying the pseudosteady-state assumption (PSSA) to this intermediate yields eq S12.

The final expression for OP_AA_, based on the quasi-Michaelis–Menten mechanism, is given as a function of Cu^2+^ concentration at time *t* as shown in eq 4 (see Text S1 for derivation)

4 $${OP}_{AA}\;\left(\mu Mh^{-1}\right)=\frac{V_{\max}{\left[{Cu}^{2+}\right]}_{t}}{K_{m}+{\left[{Cu}^{2+}\right]}_{t}}+{S\left[{Cu}^{2+}\right]}_{t}$$

The three constants, *V*_max_, *K*_*m*_, and *S*, in eq 4 are expressed in eqs 5–7, respectively, as the functions of two equilibrium constants (*K*_1_ and *K*_2_) and three reaction rate constants (*k*_1_, *k*_2_, and *k*_4_) as indicated in Scheme 1

5 $$V_{\max}=k_{4}{K_{2}\left[O_{2}\right]}_{0}$$

6 $$K_{m}=\frac{\left({k_{2}+k}_{4}\right)\left(K_{1}+1\right)}{k_{1}K_{1}}+{K_{2}\left[O_{2}\right]}_{0}$$

7 $$S=\frac{k_{3}}{\left(\frac{K_{1}+1}{K_{1}}+\frac{k_{1}K_{2}}{{k_{2}+k}_{4}}{\left[O_{2}\right]}_{0}\right)}$$

here we note that for high Cu samples at 37 °C, OP_AA_ is calculated from the initial reaction state, so [Cu^2+^]_*t*_ in eq 4 could be roughly represented by [Cu^2+^]_0_. Over longer times, if Cu^+^ oxidation is slower than its formation, progressive loss of Cu^2+^ to Cu^+^ will reduce [Cu^2+^]_*t*_, leading to a declining AA loss rate, as observed in high Cu samples (Figure 1a). In contrast, at 15 °C, the apparent zero-order kinetics suggest rapid recycling of Cu^+^ to Cu^2+^, making [Cu^2+^]_*t*_ ≈ [Cu^2+^]_0_ throughout the reaction. Thus, eq 4 simplifies to

8 $${OP}_{AA}\;\left(\mu Mh^{-1}\right)=\frac{V_{\max}{\left[{Cu}^{2+}\right]}_{0}}{K_{m}+{\left[{Cu}^{2+}\right]}_{0}}+{S\left[{Cu}^{2+}\right]}_{0}$$

Equation 8 combines a Michaelis–Menten-like term with a linear term proportional to initial Cu^2+^ concentration. Because the Michaelis–Menten term has a positive first derivative and a negative second derivative, its contribution to OP_AA_ diminishes as Cu^2+^ increases, while the linear term becomes dominant at high Cu^2+^. This explains the pronounced nonlinear trend at low Cu^2+^ and the linear increase with Cu^2+^ concentration at higher concentrations. The blue line in Figure 2b, representing a separate Michaelis–Menten regression for low Cu samples, is inaccurate because it neglects the linear term. To better verify eq 8, we subtracted the linear contribution (determined from the high Cu regression) from OP_AA_ in the low Cu samples. After this adjustment, the Michaelis–Menten regression (purple line in Figure 2b) fits the data well, supporting our derived equation. Furthermore, as shown in Figure 2c, the regression based directly on eq 8 provides an excellent fit, strongly supporting the proposed quasi-Michaelis–Menten mechanism for Cu^2+^-induced AA oxidation.

For practical OP_AA_ measurements using cuvette-type spectrometers or plate readers, the reaction time (typically a few minutes) is much shorter than in our kinetic measurement, resulting in negligible cumulative Cu^2+^ loss during the measurement. Consequently, [Cu^2+^]_*t*_ in eq 4 can be effectively approximated by the initial [Cu^2+^]_0_. Under these conditions, eq 8 can be directly applied for OP_AA_ calculation.

#### 3.1.4 Discussion of ^•^OH Formation Mechanism

Building on the quasi-Michaelis–Menten mechanism for Cu^2+^-induced AA oxidation, we note the formation rate of ^•^O_2_^–^ is the dissociation rate of the three-body intermediate, while Cu^+^ is produced via dissociation of the Cu–AA complex (see eqs S21 and S22). Both ^•^O_2_^–^ and Cu^+^ are key intermediates in the electron transfer processes that ultimately generate ^•^OH radicals. Based on previous studies, the principal reactions involving ^•^O_2_^–^ and Cu^+^ for ^•^OH formation in our system are as follows^13,25,26^

Cu++O2→j1Cu2++O2−·

O2−·+H+→j212(H2O2+O2)

H2O2+Cu+→j3OH·+OH−+Cu2+

These reactions lead to the following differential equations for the concentrations of key species

9 d[O2−·]dt=Vmax[Cu2+]0Km+[Cu2+]0+j1[O2][Cu+]−j2[H+][O2−·]

10 $$\frac{d\left[{Cu}^{+}\right]}{dt}=S{\left[{Cu}^{2+}\right]}_{0}-j_{1}\left[O_{2}\right]\left[{Cu}^{+}\right]-j_{3}\left[H_{2}O_{2}\right]\left[{Cu}^{+}\right]$$

11 d[H2O2]dt=12j2[H+][O2−·]−j3[H2O2][Cu+]

The group of equations is nonlinear and nonhomogeneous, making direct analytical solutions challenging. However, since our main interest is the ^•^OH formation rate, which is represented by *j*_3_[H_2_O_2_]­[Cu^+^], a particular solution can be derived (see eqs S21–S25 in Text S2) and shown in eq 12

12 d[OH·]dtf=13(Vmax[Cu2+]0Km+[Cu2+]0+S[Cu2+]0)=13d[AA]dt

Experimental data (Figure S1) show that cumulative ^•^OH formation follows delayed zero-order reaction kinetics (i.e., constant reaction rate independent of species at a fixed Cu^2+^ level) after a brief equilibration period, corresponding to the time required for the system to reach steady state for ^•^O_2_^–^, Cu^+^, and H_2_O_2_. Equation 12 indicates that the ^•^OH formation rate is constant over time, which is consistent with the zero-order kinetics observed in our experimental data. It also predicts that oxidation of three AA molecules yields one ^•^OH radical. However, our measurements indicate that significantly more AA is oxidized per ^•^OH formed (Figure 3a), suggesting that the electron transfer efficiency is overestimated.

**Figure 3:**
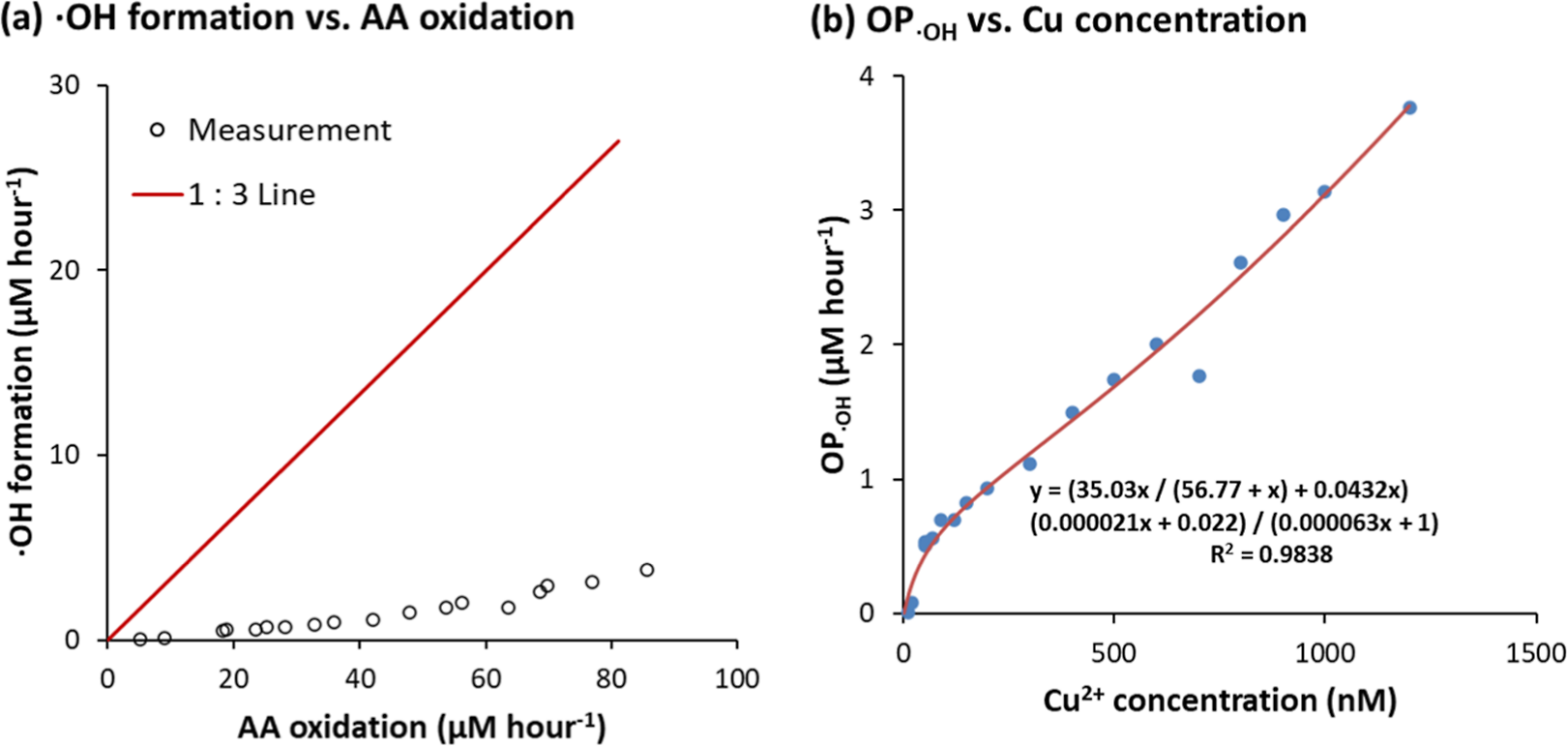
Relationships of ^•^OH formation with AA oxidation and Cu^2+^ concentration at 15 °C. (a) AA oxidation rate vs ^•^OH formation rate; (b) regression between OP_•OH_ and Cu^2+^ concentration using eq 13.

A likely explanation is the decomposition of H_2_O_2_ to H_2_O and O_2_ via dismutation, which competes with ^•^OH formation. To account for this, we include the following additional reactions

$$H_{2}O_{2}\overset{j_{4}}{\to }H_{2}O+\frac{1}{2}O_{2}$$

H2O2→j5OH·

Incorporating these reactions and following a similar derivation (eqs S26–S31), the ^•^OH formation rate can be expressed as

13 d[OH·]dtf=A[Cu2+]0+B3A[Cu2+]0+1(Vmax[Cu2+]0Km+[Cu2+]0+S[Cu2+]0)=A[Cu2+]0+B3A[Cu2+]0+1d[AA]dt

In eq 13, parameters *A* and *B* are the functions of *j*_1_, *j*_3_, *j*_4_, and *j*_5_

14 $$A=\frac{j_{3}}{2j_{4}+2j_{5}}\frac{S}{j_{1}\left[O_{2}\right]}$$

15 $$B=\frac{j_{5}}{{2j}_{4}+{2j}_{5}}$$

Equation 13 depends solely on the initial Cu^2+^ concentration, resulting in a constant ^•^OH formation rate for a fixed Cu^2+^ level. Notably, the ^•^OH formation rate is proportional to the AA oxidation rate, with the proportionality coefficient dependent on Cu^2+^. For regression analysis, the parameters *V*_max_, *K*_*m*_, and *S* were fixed to those derived from OP_AA_. As shown in Figure 3b, the regression fits the experimental data well, strongly supporting the proposed ^•^OH formation mechanism. Furthermore, according to eq S30, parameter *B* reflects the relative contribution of minor ^•^OH-producing pathways versus H_2_O_2_ dismutation. The small fitted value of *B* (0.022) from our data confirms that these minor pathways play a minimal role in ^•^OH production under our conditions.

Note that H_2_O_2_ is generated in the reaction system, but we are unable to directly quantify H_2_O_2_ in our system using HPLC coupled with a photodiode array detector (PAD). Accurate measurement of H_2_O_2_ by HPLC-PAD requires chemical derivatization (e.g., with iodide and vanillic acid to produce iodovanillic acid for detection), which would introduce unrelated species to the reaction mixture, potentially interfering with the redox cycle and affect the core OP assay. Although we currently lack experimental measurements of H_2_O_2_ and key intermediates due to these methodological constraints, the proposed mechanism allows derivation of equations to calculate their equilibrium concentrations, which can facilitate future validation once direct, nonintrusive monitoring methods become available.

### 3.2 Fe-Catalyzed AA Oxidation and ^•^OH Formation

#### 3.2.1 Fe^2+^/Fe^3+^-Induced AA Oxidation

Compared to Cu^2+^, Fe-catalyzed AA oxidation proceeds much more slowly; therefore, higher Fe concentrations were used in our experiments for the purpose of mechanistic investigation. As shown in Figure S2, cumulative AA loss increases linearly with reaction time for both Fe^2+^ and Fe^3+^ samples, indicating apparent zero-order kinetics in the AA assay. Similar to Cu^2+^, Fe^3+^ forms a three-body intermediate with AA and O_2_,^28^ suggesting that a quasi-Michaelis–Menten mechanism may also apply to Fe-induced AA oxidation. For Fe^2+^, there is no significant loss of reactive Fe at 37 °C, since Fe­(I) species are highly unstable under these conditions. Accordingly, the linear term in the quasi-Michaelis–Menten equation was omitted for Fe^2+^. In Figure 4a, excluding the anomalous drop observed at the highest two Fe^2+^ concentrations (denoted in open circles), the quasi-Michaelis–Menten model fits both Fe^2+^ and Fe^3+^ data well, supporting our mechanistic assumption (see Scheme 2). The Fe-induced OP_AA_ thus takes a similar form to that for Cu^2+^

16 $${OP}_{AA}\;\left(\mu Mh^{-1}\right)=\frac{V_{\max}{\left[Fe\right]}_{0}}{K_{m}+{\left[Fe\right]}_{0}}+{S\left[Fe\right]}_{0}$$

where *S* is zero for Fe^2+^ because Fe­(I) can hardly exist in our reaction system.

**Figure 4:**
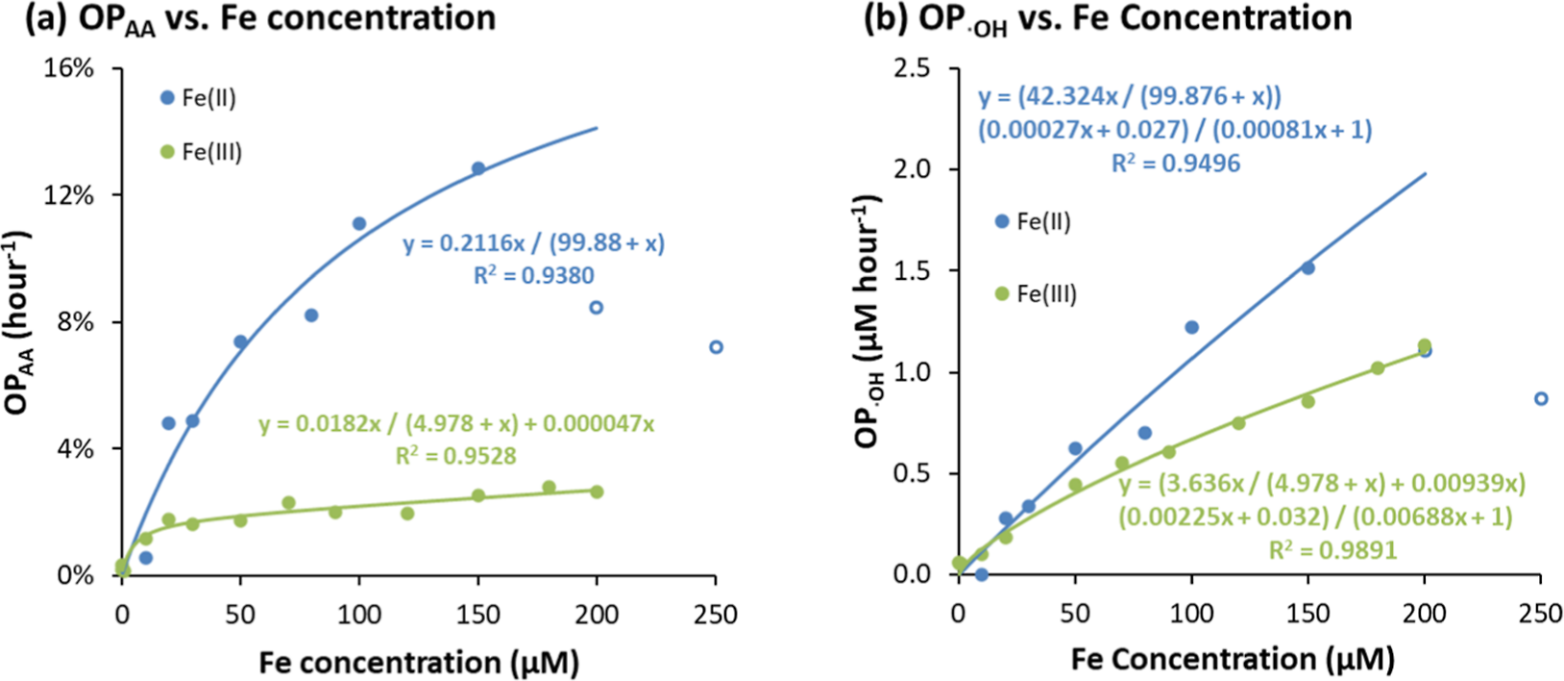
(a) Quasi-Michaelis–Menten regression between percentage OP_AA_ (h^–1^) and Fe concentration and (b) correlation between OP_•OH_ and Fe concentration.

**Scheme 2:**
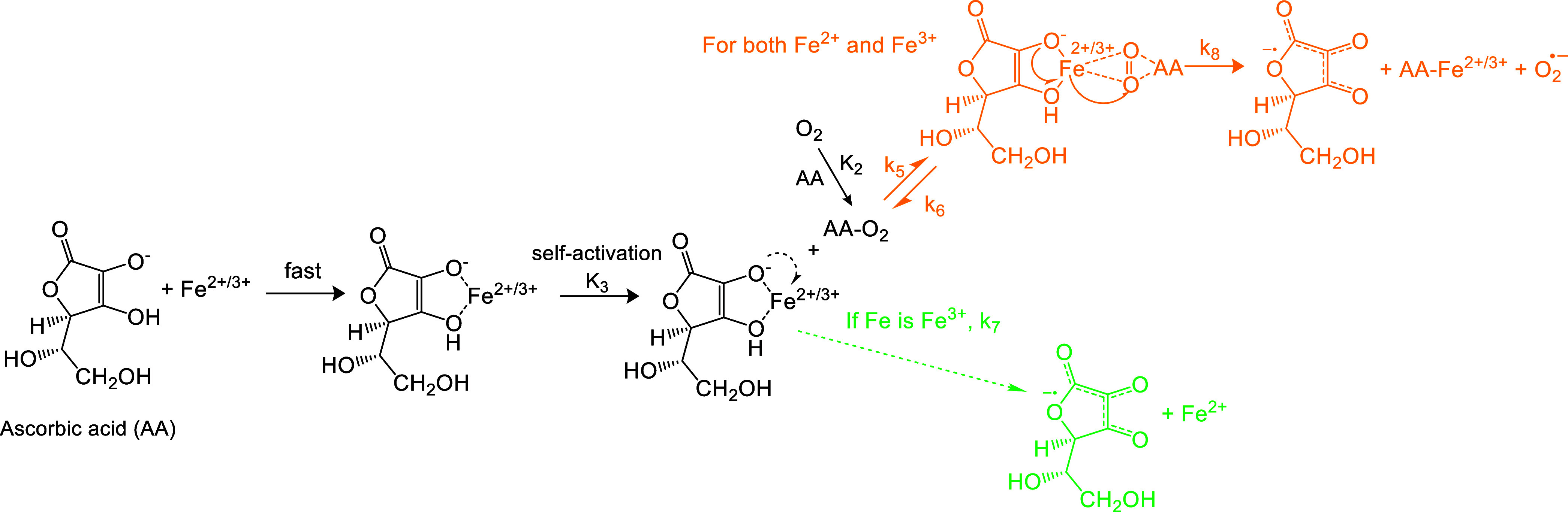
Quasi-Michaelis–Menten Mechanism for Fe-Induced AA Oxidation

The quasi-Michaelis–Menten model, however, does not explain the sudden decrease in OP_AA_ observed when Fe^2+^ exceeds 150 μM (Figure 4a). This decrease may be due to multiple factors. Note that the AA concentration (200 μM) is comparable to or lower than Fe^2+^ in the highest-dose samples, invalidating the assumption that all Fe^2+^ is rapidly complexed by AA. At high Fe^2+^/AA ratios, AA may bind multiple Fe^2+^ ions, forming larger complexes with increased steric hindrance and reduced Fe utilization. These complexes may be less effective in associating with O_2_ and facilitating electron transfer, leading to a sudden drop in Fe^2+^ induced AA loss rate in high Fe^2+^ samples.

Similar to Cu^2+^, Fe^3+^ can be reduced to Fe^2+^ during dissociation of the Fe–AA complex. Since Fe^2+^ is more effective at catalyzing AA oxidation than Fe^3+^, the Fe^2+^ pathway could, in principle, increase the AA oxidation rate in Fe^3+^ samples over time. Although the positive *S* value from regression in Figure 4a supports the existence of this Fe^2+^ pathway, no clear increase in AA loss rate over time for Fe^3+^ was observed (Figure S2b). This could be due to rapid reoxidation of Fe^2+^ to Fe^3+^ during ^•^OH formation, or because the amount of Fe^2+^ generated is negligible compared to total Fe in the system. The fitted rate constant for the Fe^2+^ pathway is 9.4 × 10^–3^ h^–1^, indicating that less than 1% of Fe^3+^ is reduced to Fe^2+^ per hour. Given concurrent Fe^2+^ reoxidation to Fe^3+^, the steady-state concentration of Fe^2+^ remains negligible relative to total Fe, which is consistent with our experimental observations for the Fe^3+^ system.

#### 3.2.2 Mechanism of ^•^OH Formation in Fe Solution

For Fe^2+^- or Fe^3+^-induced AA depletion, the influence of minor iron valence states is negligible, as confirmed by regression results (Figures 4a and S2). However, in studying ^•^OH radical formation, both Fe^2+^ and Fe^3+^ must be considered due to the Fenton reaction and Fe^2+^/Fe^3+^ redox cycling. The key Fenton-related reactions are

H2O2+Fe2+→j3OH·+OH−+Fe3+

$$H_{2}O_{2}+{Fe}^{3+}\overset{j_{6}}{\to }{HO}_{2}^{\cdot }+H^{+}+{Fe}^{2+}$$

Other relevant reactions, as in the Cu system, include

Fe2++O2→j1Fe3++O2−·

O2−·+H+→j212(H2O2+O2)

$$H_{2}O_{2}\overset{j_{4}}{\to }H_{2}O+{\frac{1}{2}O}_{2}$$

H2O2→j5OH·

These reactions are applicable for any Fe^2+^/Fe^3+^ ratio. Thus, the differential equation for ^•^O_2_^–^ can be written as

17 d[O2−·]dt=P(O2−·)+j1[O2][Fe2+]−j2[H+][O2−·]

where *P*(^•^O_2_^–^) is the ^•^O_2_^–^ production rate during Fe induced AA oxidation. Note that *P*(^•^O_2_^–^) is not a simple sum of two Michaelis–Menten terms, as there is synergy between Fe^2+^ and Fe^3+^.

The differential equations for Fe and H_2_O_2_ are

18 $$\frac{d\left[{Fe}^{2+}\right]}{dt}=-\frac{d\left[{Fe}^{3+}\right]}{dt}=S\left[{Fe}^{3+}\right]-j_{1}\left[O_{2}\right]\left[{Fe}^{2+}\right]-j_{3}\left[H_{2}O_{2}\right]\left[{Fe}^{2+}\right]+j_{6}\left[H_{2}O_{2}\right]\left[{Fe}^{3+}\right]$$

19 d[H2O2]dt=12j2[H+][O2−·]−j3[H2O2][Fe2+]−j4[H2O2]−j5[H2O2]−j6[H2O2][Fe3+]

By combining these equations and applying PSSA for ^•^O_2_^–^, Fe, and H_2_O_2_ (see Text S3), the ^•^OH formation rate can be expressed as

20 d[OH·]dtf=A[Fe2+]+B3A[Fe2+]+C[Fe3+]+1(P(O2−·)+S[Fe3+])

From Figure 4a, Fe^3+^ is much less efficient than Fe^2+^ in promoting AA depletion, and no obvious reaction rate decrease was observed over time (Figure S2a). The cumulative ^•^OH formed is much smaller than the initial Fe^2+^ concentration. These observations suggest that Fe^3+^ remains negligible in lab-prepared Fe^2+^ samples, allowing *C*[Fe^3+^] and *S*[Fe^3+^] in eq 20 to be omitted. In this case, *P*(^•^O_2_^–^) can be treated as a Michaelis–Menten-like function of Fe^2+^ concentration, simplifying eq 20 to

21 d[OH·]dtf=A[Fe2+]+B3A[Fe2+]+1(Vmax[Fe2+]Km+[Fe2+])

For lab-prepared Fe^3+^ samples (where Fe^2+^ is negligible), [Fe^2+^] can be related to [Fe^3+^] via eq 18 (see eq S37 in Text S3), yielding

22 d[OH·]dtf=A′[Fe3+]+B(3A′+C)[Fe3+]+1(Vmax[Fe3+]Km+[Fe3+]+S[Fe3+])

Similar to the Cu system (eq 13), eqs 21 and 22 show that Fe-induced ^•^OH formation rate (OP_•OH_) is proportional to OP_AA_ with a coefficient dependent on Fe^2+^/Fe^3+^ concentration. For regression, *V*_max_, *K*_*m*_, and *S* were fixed to values from the OP_AA_ fit (Figure 4a). As shown in Figure 4b, eqs 21 and 22 describe the data well for both Fe^2+^ and Fe^3+^ (*R* ^2^ > 0.9), supporting the proposed Fe-induced ^•^OH formation mechanism.

The fitted *B* value for Fe samples (∼0.03 at 37 °C) is similar, though slightly higher, than that for Cu samples (0.022 at 15 °C). As *B* reflects the proportion of minor ^•^OH-producing pathways relative to H_2_O_2_ dismutation, this difference is reasonable and likely arises from the higher reaction temperature used in Fe experiments.

### 3.3 Metal–Organics Mixture Effects in Ambient PM_2.5_ Samples

In lab-prepared Cu^2+^ solutions above 20 nM at 37 °C, a decreasing AA loss rate is clearly observed due to cumulative Cu^2+^ depletion. In contrast, water extracts of ambient PM_2.5_ samples containing >100 nM Cu^2+^ exhibit a much more linear AA loss over time under the same conditions (see Figure S3). This suggests that Cu^2+^ loss is less pronounced in ambient samples than in lab-prepared single Cu^2+^. In addition to Cu^2+^, there are other water-soluble metals and organics in ambient samples, which could be possible inhibitors to the Cu^2+^ loss process.

Theoretically, Fe has a much lower impact on AA oxidation than Cu at equivalent concentrations, as indicated by the much larger *K*_*m*_ values in the quasi-Michaelis–Menten equation. In our ambient samples, Fe concentrations were always below 1 μM, contributing negligibly to AA loss. Even experimentally, adding a much higher Fe level (2 μM) to Cu^2+^ solution did not alter the AA loss curve compared to Cu^2+^ alone (Figure S4), confirming that Fe is not responsible for the observed effect.

Ambient PM contains a complex mixture of organics, each potentially affecting TM-induced OP differently.^18^ To model real-world organics/Cu interactions, we isolated ambient organics and mixed them with the lab-prepared single metal solutions. Solid phase extraction (SPE) was used to separate ambient organics into a hydrophilic fraction (i.e., the eluted fraction), containing hydrophilic organics and most water-soluble metals, and a hydrophobic (HPO) fraction (i.e., the fraction retained on SPE and recovered using methanol rinsing), containing HULIS and only minor residual metals (Figure S5). We selected an ambient sample with relatively high Cu and HULIS and reconstituted a Cu/HPO mixture by combining its HPO fraction with a lab-prepared Cu^2+^ solution. Using paired *t*-tests at the 95% confidence level, we found that the cumulative AA loss over time differed significantly among three samples: a single Cu^2+^ solution of 100 nM, a Cu/HPO mixture solution, and a PM_2.5_ ambient water extract with a similar Cu level. As shown in Figure 5a, the AA loss in this Cu/HPO mixture remained linear over reaction time, in contrast to the nonlinear, decreasing trend observed for a single Cu^2+^ solution at the same Cu concentration (100 nM). These results demonstrate that the HULIS fraction in ambient samples alters the AA loss rate, resulting in a more sustained Cu^2+^-catalyzed oxidative effect. The slightly higher AA loss observed in the reconstituted Cu/HPO mixture, compared to the ambient extract without SPE treatment, is likely due to a minor increase in Cu content from residual metals in the HPO fraction.

**Figure 5:**
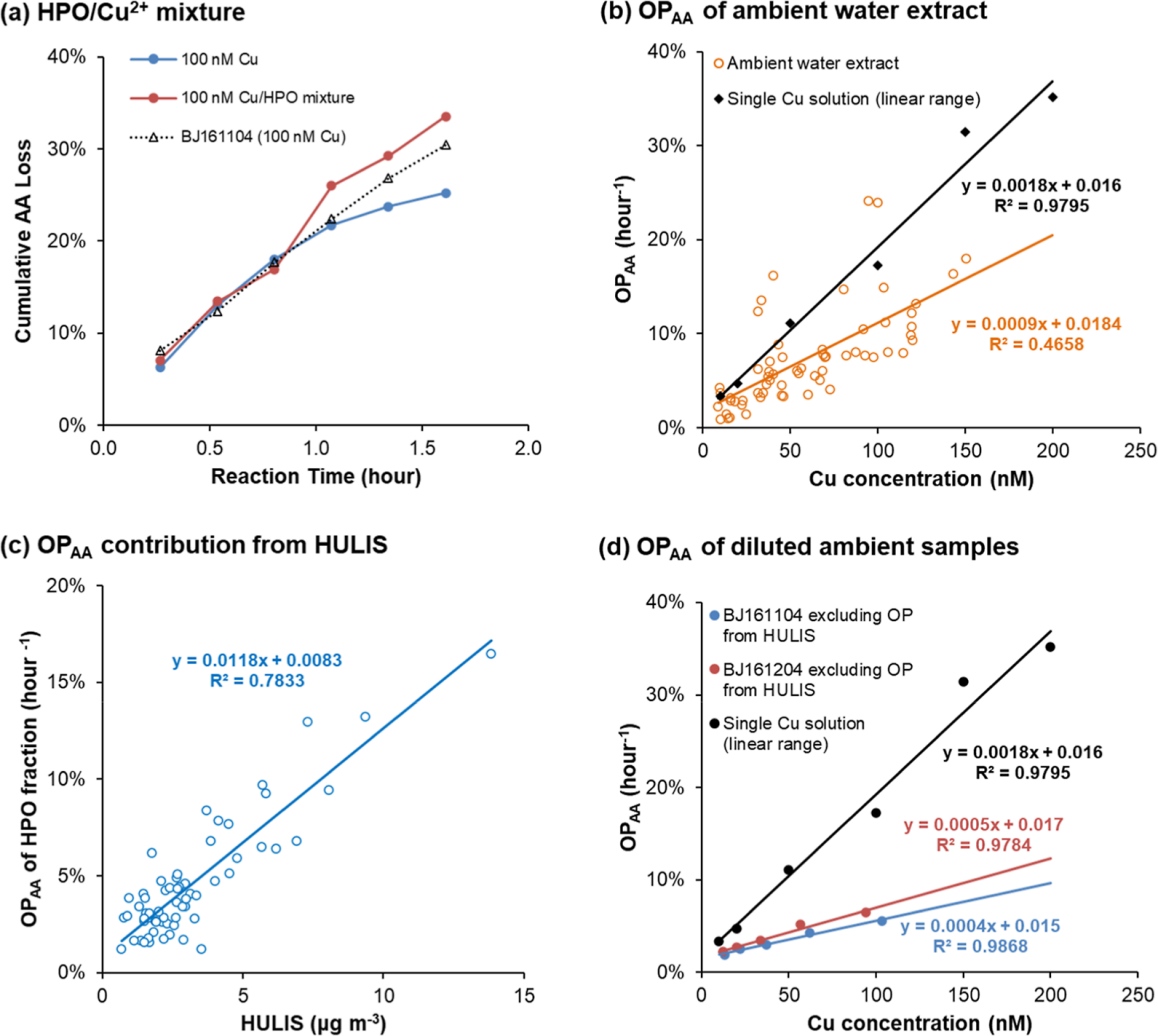
Reaction kinetics of the OP_AA_ assay and dependence of OP_AA_ on Cu or HULIS concentrations in Cu/HULIS mixtures. (a) Comparison of cumulative percentage AA loss over time for a single Cu^2+^ solution of 100 nM, an HPO/Cu mixture solution, and a PM_2.5_ ambient water extract with a similar Cu level. The ambient sample BJ161104 was collected on 4 Nov. 2016 in Beijing; (b) linear relationship between percentage OP_AA_ (h^–1^) and Cu concentration for single Cu solutions (black diamonds and line) and PM_2.5_ ambient water extracts of the Beijing 2016–2017 sample set (orange open circles and line); (c) linear relationship between percentage OP_AA_ (h^–1^) and HULIS concentration in the HPO fraction among the Beijing 2016–2017 samples; and (d) estimated Cu^2+^-induced percentage OP_AA_ (h^–1^) in two series of diluted ambient samples (red and blue dots and lines), obtained by subtracting the HULIS-induced OP_AA_ (based on the regression slope from panel c) from the total measured OP_AA_. These are compared with single Cu^2+^ solutions (black dots and line). Sample BJ161204 was collected on 4 Dec. 2016 in Beijing.

Analysis of OP_AA_ in ambient samples shows that most data points fall below the OP_AA_–Cu trend established for the lab-prepared single Cu solutions (Figure 5b). Linear regression between OP_AA_ and Cu concentration yields a lower slope for ambient samples, suggesting a reduced *S* value, likely due to the presence of HULIS in ambient water extracts. As seen from the linear OP response for HPO samples in Figure 5c, HULIS itself contributes to OP in the reaction system.

To clarify HULIS’s role in the quasi-Michaelis–Menten process, we excluded its direct OP contribution. Two representative ambient samples with high Cu and HULIS content were each diluted to five concentration levels, matching the gradients of both HULIS and Cu^2+^ across the dilution series. By subtracting the HULIS-induced OP_AA_ (estimated from Figure 5c regression) from total OP_AA_ in these diluted samples, we approximated the Cu-induced component. Since the Cu concentrations were within the linear range of the quasi-Michaelis–Menten mechanism, we applied linear regression between OP_AA_ and Cu concentration. As shown in Figure 5d, the slopes for the diluted ambient groups remained significantly lower than those for single Cu solutions, after accounting for HULIS, reinforcing the observation of a smaller *S* value in ambient samples.

Within our proposed mechanism, parameter *S* reflects the dissociation rate of the Cu–AA complex and consequent Cu^+^ formation. Thus, beyond its direct OP contribution, ambient HULIS appears to stabilize the Cu–AA intermediate, slowing cumulative Cu^2+^ loss and yielding a nearly zero-order AA oxidation process, thereby prolonging the Cu^2+^-induced OP effect at body temperature. HULIS molecules are known to bind TM ions;^18,19,23^ their oxygen- and nitrogen-containing functional groups may also form hydrogen bonds with AA. These binding properties likely underpin the stabilizing effect of HULIS in the quasi-Michaelis–Menten reaction process.

## 4 Implications and Future Perspectives

This study provides new insights into the reaction kinetics underlying Cu^2+^-induced AA oxidation in laboratory systems. We observed a unique kinetic behavior of the OP_AA_ assay reaction at body temperature that cannot be explained by either the classical zero-order Michaelis–Menten or second-order kinetics. By conducting experiments at 15 °C, we revealed a transition from nonlinear to linear dependence of AA loss rate on Cu^2+^ concentration, which is well described by a quasi-Michaelis–Menten mechanism initiated by a Cu^2+^–AA intermediate. This intermediate follows two primary pathways: reaction with oxygen, analogous to the original Michaelis–Menten reaction in the substrate–enzyme system, and direct dissociation yielding oxidized AA and Cu^+^. The balance between these pathways governs whether AA oxidation follows zero-order kinetics or exhibits a declining rate due to cumulative Cu^2+^ loss.

We further elucidated the mechanism of ^•^OH formation, demonstrating that the process is closely linked to the fate of Cu^+^ and ^•^O_2_^–^ generated during Cu^2+^-catalyzed AA oxidation. The derived rate equations accurately fit experimental data and the data suggest that electron transfer from AA to ^•^OH is not 100% due to H_2_O_2_ decomposition. While Fe induces much lower OP compared to Cu^2+^, similar quasi-Michaelis–Menten kinetics and ^•^OH formation mechanisms apply to Fe^2+^/Fe^3+^ within certain concentration ranges.

In ambient PM_2.5_ extracts at 37 °C, the decrease in AA oxidation rate attributable to Cu^2+^ loss is less pronounced than in pure Cu^2+^ solutions. Our findings indicate that Fe has minimal impact on overall OP generation at ambient levels, whereas ambient HULIS evidently delays the decline in AA oxidation rate with reaction time. HULIS not only directly contributes to OP but also inhibits dissociation of the Cu^2+^–AA intermediate, likely owing to its strong binding affinities for both AA and Cu^2+^.

Some kinetic parameters derived from the lab-prepared single metal solutions are shown in Table S1 as a general reference. However, these parameters can vary significantly depending on the sample matrix in ambient aerosols. Therefore, we recommend performing dilutions for each ambient sample to determine the most accurate kinetic parameters.

The quasi-Michaelis–Menten framework presented here offers a quantitative chemical basis for relating TM concentrations to OP in the AA assay and clarifies the previously underappreciated role of ambient HULIS. These mechanistic insights provide a foundation for improved OP modeling and interpretation based on ambient PM composition and highlight the need for further research into the complex interactions among TMs, organics, and ROS in atmospheric particulate matter.

## Supplementary Material



## Notes

Additional details for reaction mechanisms and derivations of rate expressions for ascorbic acid oxidation and hydroxyl radical formation; additional figures presenting kinetic experiment results; and kinetic coefficients calculated using laboratory data in this study (PDF)

## References

[1] Churg A., Brauer M. (1997). Human lung parenchyma retains PM2.5. Am. J. Respir. Crit. Care Med..

[2] Pinkerton K. E., Green F. H. Y., Saiki C., Vallyathan V., Plopper C. G., Gopal V., Hung D., Bahne E. B., Lin S. S., Ménache M. G., Schenker M. B. (2000). Distribution of
Particulate Matter and Tissue Remodeling in the Human Lung. Environ. Health Perspect..

[3] Feng S. L., Gao D., Liao F., Zhou F. R., Wang X. M. (2016). The health effects
of ambient Pm2.5 and potential mechanisms. Ecotoxicol.
Environ. Saf..

[4] Cui Y. Q., Xie X. Y., Jia F. P., He J. F., Li Z. H., Fu M. H., Hao H., Liu Y., Liu J. Z., Cowan P. J., Zhu H., Sun Q. H., Liu Z. G. (2015). Ambient
Fine Particulate Matter Induces Apoptosis of Endothelial Progenitor
Cells Through Reactive Oxygen Species Formation. Cell. Physiol. Biochem..

[5] Cho C. C., Hsieh W. Y., Tsai C. H., Chen C. Y., Chang H. F., Lin C. S. (2018). In vitro and in vivo experimental studies of Pm2.5
on disease progression. Int. J. Environ. Res.
Public Health.

[6] Valavanidis A., Vlachogianni T., Fiotakis K., Loridas S. (2013). Pulmonary Oxidative
Stress, Inflammation and Cancer: Respirable Particulate Matter, Fibrous
Dusts and Ozone as Major Causes of Lung Carcinogenesis through Reactive
Oxygen Species Mechanisms. Int. J. Environ.
Res. Public Health.

[7] Zhong C. Y., Zhou Y. M., Smith K. R., Kennedy I. M., Chen C. Y., Aust A. E., Pinkerton K. E. (2010). Oxidative injury in the lungs of
neonatal rats following short-term exposure to ultrafine iron and
soot particles. J. Toxicol. Environ. Health,
Part A.

[8] Cross C. E., van der Vliet A., O’Neill C. A., Louie S., Halliwell B. (1994). Oxidants,
Antioxidants, and Respiratory-Tract Lining Fluids. Environ. Health Perspect..

[9] Das K., Roychoudhury A. (2014). Reactive oxygen species (ROS) and response of antioxidants
as ROS-scavengers during environmental stress in plants. Front. Environ. Sci..

[10] Noctor G. (2006). Metabolic
signalling in defence and stress: the central roles of soluble redox
couples. Plant, Cell Environ..

[11] Cho A. K., Sioutas C., Miguel A. H., Kumagai Y., Schmitz D. A., Singh M., Eiguren-Fernandez A., Froines J. R. (2005). Redox activity of
airborne particulate matter at different sites in the Los Angeles
Basin. Environ. Res..

[12] Charrier J. G., Anastasio C. (2015). Rates of hydroxyl
radical production from transition
metals and quinones in a surrogate lung fluid. Environ. Sci. Technol..

[13] DiStefano E., Eiguren-Fernandez A., Delfino R. J., Sioutas C., Froines J. R., Cho A. K. (2009). Determination of metal-based hydroxyl radical generating
capacity of ambient and diesel exhaust particles. Inhalation Toxicol..

[14] Fang T., Verma V., Bates J. T., Abrams J., Klein M., Strickland M. J., Sarnat S. E., Chang H. H., Mulholland J. A., Tolbert P. E., Russell A. G., Weber R. J. (2016). Oxidative
potential
of ambient water-soluble PM2.5 in the southeastern United States:
contrasts in sources and health associations between ascorbic acid
(AA) and dithiothreitol (DTT) assays. Atmos.
Chem. Phys..

[15] Vidrio E., Jung H., Anastasio C. (2008). Generation
of hydroxyl radicals from
dissolved transition metals in surrogate lung fluid solutions. Atmos. Environ..

[16] Visentin M., Pagnoni A., Sarti E., Pietrogrande M. C. (2016). Urban PM2.5
oxidative potential: Importance of chemical species and comparison
of two spectrophotometric cell-free assays. Environ. Pollut..

[17] Lin M. F., Yu J. Z. (2019). Effect of Metal-organic
Interactions on the Oxidative Potential of
Mixtures of Atmospheric Humic-like Substances and Copper/Manganese
as Investigated by the Dithiothreitol Assay. Sci. Total Environ..

[18] Lin M. F., Yu J. Z. (2020). Assessment of Interactions
between Transition Metals and Atmospheric
Organics: Ascorbic Acid Depletion and Hydroxyl Radical Formation in
Organic-Metal Mixtures. Environ. Sci. Technol..

[19] Lin P., Yu J. Z. (2011). Generation of Reactive
Oxygen Species Mediated by Humic-like Substances
in Atmospheric Aerosols. Environ. Sci. Technol..

[20] Verma V., Wang Y., El-Afifi R., Fang T., Rowland J., Russell A. G., Weber R. J. (2015). Fractionating
ambient humic-like
substances (HULIS) for their reactive oxygen species activity –
Assessing the importance of quinones and atmospheric aging. Atmos. Environ..

[21] Charrier J. G., Anastasio C. (2012). On dithiothreitol
(DTT) as a measure of oxidative potential
for ambient particles: evidence for the importance of soluble transition
metals. Atmos. Chem. Phys..

[22] Charrier J. G., Mcfall A. S., Vu K. K., Baroi J., Olea C., Hasson A., Anastasio C. (2016). A bias in
the “mass-normalized”
DTT response – An effect of non-linear concentration-response
curves for copper and manganese. Atmos. Environ..

[23] Lin M. F., Yu J. Z. (2019). Dithiothreitol (DTT)
Concentration Effect and its Implications on
the Applicability of DTT Assay to Evaluate the Oxidative Potential
of Atmospheric Aerosol Samples. Environ. Pollut..

[24] Gao D., Godri Pollitt K. J., Mulholland J. A., Russell A. G., Weber R. J. (2020). Characterization
and comparison of PM2.5 oxidative potential assessed by two acellular
assays. Atmos. Chem. Phys..

[25] Scarpa M., Momo F., Viglino P., Vianello F., Rigo A. (1996). Activated
oxygen species in the oxidation of glutathione A kinetic study. Biophys. Chem..

[26] Yuan X., Pham A. N., Xing G., Rose A. L., Waite T. D. (2012). Effects
of pH, Chloride, and Bicarbonate on Cu­(I) Oxidation Kinetics at Circumneutral
pH. Environ. Sci. Technol..

[27] Buettner G. R. (1988). In the
absence of catalytic metals ascorbate does not autoxidize at pH 7:
ascorbate as a test for catalytic metals. J.
Biochem. Biophys. Methods.

[28] Khan M. M. T., Martell A. E. (1967). Metal ion and metal chelate catalyzed
oxidation of
ascorbic acid by molecular oxygen. I. Cupric and ferric ion catalyzed
oxidation. J. Am. Chem. Soc..

[29] Jameson R. F., Blackburn N. J. (1976). Role of copper dimers and the participation
of copper
(III) in the copper-catalysed autoxidation of ascorbic acid. Part
III. Kinetics and mechanism in 0.100 mol dm–3 potassium chloride. J. Chem. Soc., Dalton Trans..

[30] Shtamm E., Purmal A., Skurlatov Y. I. (1979). Mechanism of catalytic ascorbic acid
oxidation system Cu^2+^–ascorbic acid–O_2_. Int. J. Chem. Kinet..

[31] Charrier J. G., Anastasio C. (2011). Impacts of
Antioxidants on Hydroxyl Radical Production
from Individual and Mixed Transition Metals in a Surrogate Lung Fluid. Atmos. Environ..

[32] Shen H. Y., Anastasio C. (2012). A comparison
of hydroxyl radical and hydrogen peroxide
generation in ambient particle extracts and laboratory metal solutions. Atmos. Environ..

[33] Hayakawa K., Hayashi Y. (1977). Detection of a complex
intermediate in the oxidation
of ascorbic acid by the copper (II) ion. J.
Nutr. Sci. Vitaminol..

[34] Mahata S., Mitra I., Mukherjee S., Reddy V. P. B., Ghosh G. K., Wolfgang W., Moi S. C. (2019). Speciation Study of L-ascorbic Acid
and its Chelated Cu (II) & Ni (II) Complexes: an Experimental
and Theoretical Model of Complex Formation. S. Afr. J. Chem..

[35] Wilson R. J., Beezer A. E., Mitchell J. C. (1995). A kinetic
study of the oxidation
of L-ascorbic acid (vitamin C) in solution using an isothermal microcalorimeter. Thermochim. Acta.

