# Supplementary material for: Linking Transition Metal Concentration and Oxidative Potential in PM2.5 by Ascorbic Acid Assay via Quasi-Michaelis–Menten Mechanism
Source: Environ Sci Technol. 2025 Oct 21;59(43):23161–72. doi: 10.1021/acs.est.5c09374 (PMC12593405; doi:10.1021/acs.est.5c09374)
Supplement: Supplementary file 1 [file es5c09374_si_001.pdf]

Supporting Information

Linking Transition Metal Concentration and Oxidative Potential in $PM_{2.5}$ by Ascorbic Acid Assay via Quasi-Michaelis–Menten Mechanism

Yuhuang Cheng[1], Hanzhe Chen[2], and Jian Zhen Yu[1,2,*]

[1]Department of Chemistry, Hong Kong University of Science & Technology, Hong Kong 999077, China

[2]Division of Environment and Sustainability, Hong Kong University of Science & Technology, Hong Kong 999077, China

*Corresponding author: chjianyu@ust.hk

This file contains

Three supporting text sections

Five supporting figures

One supporting table

Part A Supporting text sections

Text S1. Reaction rate calculation for the quasi-Michaelis–Menten mechanism of the Cu^{2+}-induced AA oxidation

Before discussing the quasi-Michaelis–Menten mechanism, we would like to briefly introduce the classical Michaelis–Menten mechanism. In the binary enzyme–substrate system, the enzyme E and substrate S first form an enzyme–substrate intermediate (E–S in Scheme S1). The forward and reverse rate constant of this step are represented as k_+ and k_-. It is assumed that there is pseudo-steady-state for E–S, which means that E–S concentration remains unchanged during the reaction. The intermediate E–S then dissociates into product P and enzyme E with rate constant k_{cat}.

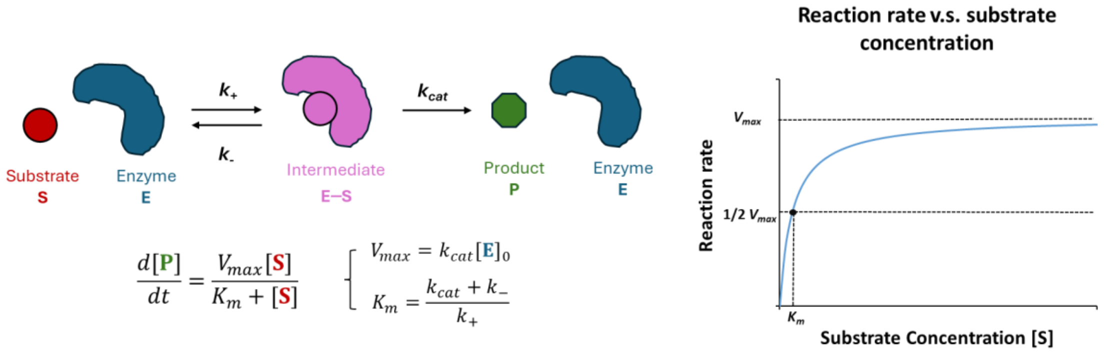

Scheme S1. Classical Michaelis–Menten mechanism in the binary enzyme–substrate system.

Based on the mechanism, we can have the following differential equation for E–S concentration:

$$\frac{d[\mathrm{E-S}]}{dt} = k_+[\mathrm{E}][\mathrm{S}] - (k_- + k_{cat})[\mathrm{E-S}] = 0 \qquad \text{(S1)}$$

The conservation relationship of enzyme E is written as:

$$[\mathrm{E}]_0 = [\mathrm{E-S}] + [\mathrm{E}] \qquad \text{(S2)}$$

From equations S1 and S2, after cancelling out [E–S], the real-time concentration of enzyme E is represented by its initial concentration $[\mathrm{E}]_0$:

$$[\mathrm{E}] = [\mathrm{E}]_0 \frac{k_- + k_{cat}}{k_- + k_{cat} + k_+[\mathrm{S}]} \qquad \text{(S3)}$$

Then, substitute eq S3 into eq S2, concentration of intermediate E–S could be expressed as follows:

$$[\mathrm{E-S}] = \frac{[\mathrm{E}]_0[\mathrm{S}]}{\frac{k_- + k_{cat}}{k_+} + [\mathrm{S}]} \qquad \text{(S4)}$$

The formation rate of product P is proportional to [E–S]:

$$\frac{d[\mathrm{P}]}{dt} = k_{cat}[\mathrm{E-S}] = \frac{k_{cat}[\mathrm{E}]_0[\mathrm{S}]}{\frac{k_- + k_{cat}}{k_+} + [\mathrm{S}]} = \frac{V_{max}[\mathrm{S}]}{K_m + [\mathrm{S}]} \quad \text{(S5)}$$

Where K_m and V_{max} are the two key parameters of the classical Michaelis–Menten mechanism, which could be represented by several constants:

$$K_m = \frac{k_- + k_{cat}}{k_+} \quad \text{(S6)}$$

$$V_{max} = k_{cat}[\mathrm{E}]_0 \quad \text{(S7)}$$

Plotting the formation rate of P over substrate concentration based on eq S5, we could observe a nonlinear curve, shown in the right part of Scheme S1. The reaction rate increases with S concentration, and such increase slows down with increasing S concentration. This makes the curve almost horizontal to the x-axis in high substrate concentration region. V_{max} is the theoretical maximum reaction rate when substrate concentration approaches infinity, while K_m is the substrate value at which the reaction rate reaches half of the V_{max} value:

$$\lim_{[\mathrm{S}]\to\infty} \frac{V_{max}[\mathrm{S}]}{K_m + [\mathrm{S}]} = V_{max} \quad \text{(S8)}$$

$$\left(\frac{d[\mathrm{P}]}{dt}\right)_{[\mathrm{S}]=K_m} = \frac{1}{2}V_{max} \quad \text{(S9)}$$

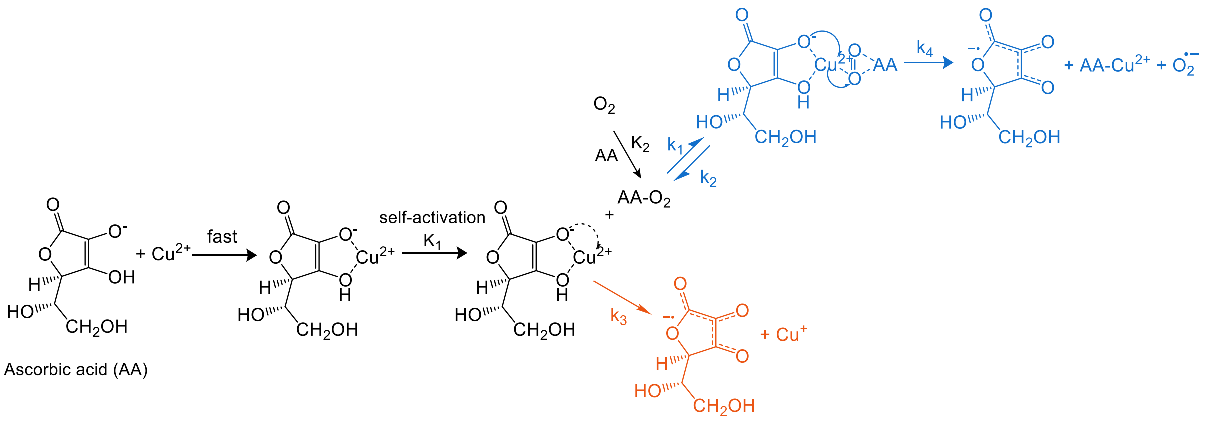

Scheme 1. Quasi-Michaelis–Menten mechanism for Cu^{2+}-induced AA oxidation.

For Cu-induced AA oxidation in AA assay, the reaction initiated with fast formation of a Cu–AA complex, followed by a self-activation process:

$$K_1[\mathrm{Cu}^{2+} - AA] = [\mathrm{Cu}^{(2-\delta)+} - \mathrm{AA}^{\delta+}] \quad \text{(S10)}$$

AA can also be directly oxidized by dissolved oxygen in the absence of metal ions in aqueous systems. One previous kinetic study indicates that this reaction is first order with respect to oxygen, but

independent of AA concentration.[1] If AA–O_2 is considered an intermediate, its concentration should be proportional to dissolved oxygen, defined by a constant K_2:

$$K_2[\mathrm{O_2}] = [\mathrm{AA-O_2}] \quad \text{(S11)}$$

We have PSSA for the three-body intermediate:

$$k_1\left[\mathrm{Cu^{(2-\delta)+}-AA^{\delta+}}\right][\mathrm{AA-O_2}] = (k_2+k_4)[\mathrm{AA-O_2-Cu^{(2-\delta)+}-AA^{\delta+}}] \quad \text{(S12)}$$

For easier expression, we consider the Cu in the intermediates as Cu^{2+}, then we have the following conservative relationship for Cu:

$$[\mathrm{Cu^{2+}}]_t = [\mathrm{Cu^{2+}}]_0 - [\mathrm{Cu^{+}}]_t = [\mathrm{Cu^{2+}-AA}] + \left[\mathrm{Cu^{(2-\delta)+}-AA^{\delta+}}\right] + \left[\mathrm{AA-O_2-Cu^{(2-\delta)+}-AA^{\delta+}}\right] \quad \text{(S13)}$$

Putting equations S10–S12 into S13, we can get the following relationship:

$$[\mathrm{Cu^{2+}}]_t = [\mathrm{Cu^{2+}}]_0 - [\mathrm{Cu^{+}}]_t = \left(\frac{K_1+1}{K_1} + \frac{k_1}{k_2+k_4}[\mathrm{AA-O_2}]\right)\left[\mathrm{Cu^{(2-\delta)+}-AA^{\delta+}}\right] \quad \text{(S14)}$$

For oxygen, we assumed that the consumed oxygen in the reaction would be quickly replenished by the gaseous oxygen above the surrogate lung fluid, then we can get:

$$K_2[\mathrm{O_2}]_0 = [\mathrm{AA-O_2}]_0 = [\mathrm{AA-O_2}] + \left[\mathrm{AA-O_2-Cu^{(2-\delta)+}-AA^{\delta+}}\right] \quad \text{(S15)}$$

Putting eq S12 into S15, we can get:

$$K_2[\mathrm{O_2}]_0 = \left(1 + \frac{k_2+k_4}{k_1\left[\mathrm{Cu^{(2-\delta)+}-AA^{\delta+}}\right]}\right)\left[\mathrm{AA-O_2-Cu^{(2-\delta)+}-AA^{\delta+}}\right] \quad \text{(S16)}$$

The total AA oxidation rate could be expressed as:

$$\frac{d[\mathrm{AA_{oxi}}]}{dt} = \mathrm{OP_{AA}}(\mu\mathrm{M\ h^{-1}}) = [\mathrm{AA}]_0\mathrm{OP_{AA}}(\%, \mathrm{h^{-1}}) = \frac{d[\mathrm{AA_{oxi}}]}{dt}_{\left(\mathrm{Cu^{(2-\delta)+}-AA^{\delta+}}\right)} + \frac{d[\mathrm{AA_{oxi}}]}{dt}_{\left(\mathrm{O_2-Cu^{(2-\delta)+}-AA^{\delta+}}\right)}$$

$$\frac{d[\mathrm{AA_{oxi}}]}{dt} = \mathrm{OP_{AA}}(\mu\mathrm{M\ h^{-1}}) = k_3\left[\mathrm{Cu^{(2-\delta)+}-AA^{\delta+}}\right] + k_4[\mathrm{AA-O_2-Cu^{(2-\delta)+}-AA^{\delta+}}] \quad \text{(S17)}$$

Putting equations S14 and S16 into S17, we got the final expression of OP_{AA}:

$$\mathrm{OP_{AA}}(\mu\mathrm{M\ h^{-1}}) = k_4K_2[\mathrm{O_2}]_0\frac{([\mathrm{Cu^{2+}}]_0-[\mathrm{Cu^{+}}]_t)}{\frac{(k_2+k_4)(K_1+1)}{k_1K_1}+[\mathrm{AA-O_2}]+([\mathrm{Cu^{2+}}]_0-[\mathrm{Cu^{+}}]_t)} + \frac{k_3([\mathrm{Cu^{2+}}]_0-[\mathrm{Cu^{+}}]_t)}{\frac{K_1+1}{K_1}+\frac{k_1}{k_2+k_4}[\mathrm{AA-O_2}]}$$

$$\mathrm{OP_{AA}}(\mu\mathrm{M\ h^{-1}}) = k_4K_2[\mathrm{O_2}]_0\frac{[\mathrm{Cu^{2+}}]_t}{\frac{(k_2+k_4)(K_1+1)}{k_1K_1}+[\mathrm{AA-O_2}]+[\mathrm{Cu^{2+}}]_t} + \frac{k_3[\mathrm{Cu^{2+}}]_t}{\frac{K_1+1}{K_1}+\frac{k_1}{k_2+k_4}[\mathrm{AA-O_2}]} \quad \text{(S18)}$$

We assumed that the three-body intermediate has negligible concentration compared with AA–O_2:

$$\mathrm{OP_{AA}}(\mu\mathrm{M\ h^{-1}}) = k_4K_2[\mathrm{O_2}]_0\frac{[\mathrm{Cu^{2+}}]_t}{\frac{(k_2+k_4)(K_1+1)}{k_1K_1}+K_2[\mathrm{O_2}]_0+[\mathrm{Cu^{2+}}]_t} + \frac{k_3[\mathrm{Cu^{2+}}]_t}{\frac{K_1+1}{K_1}+\frac{k_1K_2}{k_2+k_4}[\mathrm{O_2}]_0} \quad \text{(S19)}$$

The final expression of OP_{AA} could be simply written in the form of:

$$\mathrm{OP_{AA}}(\mu\mathrm{M\ h^{-1}}) = [\mathrm{AA}]_0\mathrm{OP_{AA}}(\%, \mathrm{h^{-1}}) = \frac{V_{max}[\mathrm{Cu^{2+}}]_t}{K_m + [\mathrm{Cu^{2+}}]_t} + S[\mathrm{Cu^{2+}}]_t \quad \text{(S20)}$$

Where the three parameters could be represented by several constants:

$$V_{max} = k_4 K_2 [\mathrm{O_2}]_0$$

$$K_m = \frac{(k_2 + k_4)(K_1 + 1)}{k_1 K_1} + K_2[\mathrm{O_2}]_0$$

$$S = \frac{k_3}{(\frac{K_1 + 1}{K_1} + \frac{k_1 K_2}{k_2 + k_4}[\mathrm{O_2}]_0)}$$

Remember we include the AA–O_2 intermediate shown by eq S11, if we directly use dissolved O_2 instead of this AA–O_2 intermediate, we can still get the similar expression of $\mathrm{OP_{AA}}$, with a slightly different expression of K_m for the Michaelis–Menten part:

$$K_m = \frac{(k_2 + k_4)(K_1 + 1)}{k_1 K_1} + [\mathrm{O_2}]_0 > [\mathrm{O_2}]_0$$

Seen from Fig. 2c, the K_m value from the regression result is 56.77 nM, which is much smaller than the theoretical K_m if the hypothesis is not applied (the dissolved oxygen is around 200 μM). This contradiction comes from the $[O_2]_0$ in K_m, which is introduced by [O_2–$Cu^{(2-\delta)+}$–$AA^{\delta+}$]:

$$\left[\mathrm{O_2} - \mathrm{Cu^{(2-\delta)+}} - \mathrm{AA^{\delta+}}\right] = \left(\frac{k_1}{k_2 + k_4}[\mathrm{O_2}]\right)\left[\mathrm{Cu^{(2-\delta)+}} - \mathrm{AA^{\delta+}}\right]$$

The excessively high K_m value equally means an overestimated concentration of the three-body intermediate compared with the binary intermediate. From this perspective, the activated oxygen hypothesis avoids the contradiction by introducing a small K_2, which gives a much lower [AA–O_2–$Cu^{(2-\delta)+}$–$AA^{\delta+}$], both chemically and mathematically.

Text S2. Differential equation solution for Cu induced ·OH formation process

The formation rate of $\cdot O_2^-$ and Cu^+ caused by AA oxidation are written as follows:

$$\frac{d[\cdot O_2^-]}{dt}_{AA} = \frac{d[AA_{oxi}]}{dt}_{\left(O_2-Cu^{(2-\delta)+}-AA^{\delta+}\right)} = \frac{V_{max}[Cu^{2+}]_0}{K_m+[Cu^{2+}]_0} \quad (S21)$$

$$\frac{d[Cu^+]}{dt}_{AA} = \frac{d[AA_{oxi}]}{dt}_{\left(Cu^{(2-\delta)+}-AA^{\delta+}\right)} = S[Cu^{2+}]_0 \quad (S22)$$

Reactions for ·OH formation process:

$$Cu^+ + O_2 \xrightarrow{j_1} Cu^{2+} + \cdot O_2^-$$

$$\cdot O_2^- + H^+ \xrightarrow{j_2} \frac{1}{2}(H_2O_2 + O_2)$$

$$H_2O_2 + Cu^+ \xrightarrow{j_3} \cdot OH + OH^- + Cu^{2+}$$

The three differential equations for ·OH formation process:

$$\frac{d[\cdot O_2^-]}{dt} = \frac{V_{max}[Cu^{2+}]_0}{K_m+[Cu^{2+}]_0} + j_1[O_2][Cu^+] - j_2[H^+][\cdot O_2^-] \quad (9)$$

$$\frac{d[Cu^+]}{dt} = S[Cu^{2+}]_0 - j_1[O_2][Cu^+] - j_3[H_2O_2][Cu^+] \quad (10)$$

$$\frac{d[H_2O_2]}{dt} = \frac{1}{2}j_2[H^+][\cdot O_2^-] - j_3[H_2O_2][Cu^+] \quad (11)$$

Linearly combining the three differential equations by eq 9 + eq 10 + 2*eq 11:

$$\frac{d[\cdot O_2^-]}{dt} + \frac{d[Cu^+]}{dt} + 2\frac{d[H_2O_2]}{dt} = \frac{V_{max}[Cu^{2+}]_0}{K_m+[Cu^{2+}]_0} + S[Cu^{2+}]_0 - 3j_3[H_2O_2][Cu^+] \quad (S23)$$

The ·OH formation rate we care about is $j_3[H_2O_2][Cu^+]$:

$$\frac{d[\cdot OH]}{dt}_f = j_3[H_2O_2][Cu^+] = \frac{1}{3}\left(\frac{V_{max}[Cu^{2+}]_0}{K_m+[Cu^{2+}]_0} + S[Cu^{2+}]_0 - \left(\frac{d[\cdot O_2^-]}{dt} + \frac{d[Cu^+]}{dt} + 2\frac{d[H_2O_2]}{dt}\right)\right) \quad (S24)$$

When we apply PSSA for $\cdot O_2^-$, Cu^+, and H_2O_2 ($\frac{d[\cdot O_2^-]}{dt} = \frac{d[Cu^+]}{dt} = \frac{d[H_2O_2]}{dt} = 0$):

$$\frac{d[\cdot OH]}{dt}_f = j_3[H_2O_2][Cu^+] = \frac{1}{3}\left(\frac{V_{max}[Cu^{2+}]_0}{K_m+[Cu^{2+}]_0} + S[Cu^{2+}]_0\right) = \frac{1}{3}\frac{d[AA]}{dt} = \frac{1}{3}[AA]_0 OP_{AA} \quad (S25)$$

Considering the H_2O_2 decomposition reactions and other minor chemical processes that H_2O_2 produces ·OH:

$$Cu^+ + O_2 \xrightarrow{j_1} Cu^{2+} + \cdot O_2^-$$

$$\cdot O_2^- + H^+ \xrightarrow{j_2} \frac{1}{2}(H_2O_2 + O_2)$$

$$H_2O_2 + Cu^+ \xrightarrow{j_3} \cdot OH + OH^- + Cu^{2+}$$

$$H_2O_2 \xrightarrow{j_4} H_2O + \frac{1}{2}O_2$$

$$H_2O_2 \xrightarrow{j_5} \cdot OH$$

The three differential equations for ·OH formation process could be rewritten as:

$$\frac{d[\cdot O_2^-]}{dt} = \frac{V_{max}[Cu^{2+}]_0}{K_m+[Cu^{2+}]_0} + j_1[O_2][Cu^+] - j_2[H^+][\cdot O_2^-] \quad (9)$$

$$\frac{d[Cu^+]}{dt} = S[Cu^{2+}]_0 - j_1[O_2][Cu^+] - j_3[H_2O_2][Cu^+] \quad (10)$$

$$\frac{d[H_2O_2]}{dt} = \frac{1}{2}j_2[H^+][\cdot O_2^-] - j_3[H_2O_2][Cu^+] - j_4[H_2O_2] - j_5[H_2O_2] \quad (11)$$

Linearly combining the three differential equations by eq 9 + eq 10 + 2*eq 11:

$$\frac{d[\cdot O_2^-]}{dt} + \frac{d[Cu^+]}{dt} + \frac{2d[H_2O_2]}{dt} = \frac{V_{max}[Cu^{2+}]_0}{K_m+[Cu^{2+}]_0} + S[Cu^{2+}]_0 - (3j_3[Cu^+] + 2j_4 + 2j_5)[H_2O_2] \quad (S26)$$

Applying PSSA for $\cdot O_2^-$, Cu^+, and H_2O_2 ($\frac{d[\cdot O_2^-]}{dt} = \frac{d[Cu^+]}{dt} = \frac{d[H_2O_2]}{dt} = 0$):

$$(3j_3[Cu^+] + 2j_4 + 2j_5)[H_2O_2] = \frac{V_{max}[Cu^{2+}]_0}{K_m+[Cu^{2+}]_0} + S[Cu^{2+}]_0 \quad (S27)$$

The ·OH formation rate we care about is $j_3[H_2O_2][Cu^+] + j_5[H_2O_2]$:

$$\left.\frac{d[\cdot OH]}{dt}\right|_f = j_3[H_2O_2][Cu^+] + j_5[H_2O_2] = \frac{j_3[Cu^+]+j_5}{3j_3[Cu^+]+2j_4+2j_5}\frac{d[AA]}{dt} \quad (S28)$$

Because of the PSSA for Cu^+, we can express Cu^+ by Cu^{2+}:

$$[Cu^+] = \frac{S[Cu^{2+}]_0}{j_1[O_2]+j_3[H_2O_2]} \approx \frac{S[Cu^{2+}]_0}{j_1[O_2]} \quad (S29)$$

Since O_2 is much more abundant than H_2O_2 in the system, we applied this approximation in eq S29. In addition, we need to emphasize that the nonzero Cu^+ concentration has no contradiction with the negligible Cu^{2+} loss we assumed for AA oxidation step under 15°C. When putting the j_1 value (3.1×10^4 M^{-1} s^{-1}) from the previous research[2] into eq S29, the estimated Cu^+/Cu^{2+} ratio is only around 0.002, in which case the negligible Cu^{2+} loss is still valid. Putting eq S29 into S28:

$$\left.\frac{d[\cdot OH]}{dt}\right|_f = \frac{\frac{j_3}{2j_4+2j_5}\frac{S[Cu^{2+}]_0}{j_1[O_2]}+\frac{j_5}{2j_4+2j_5}}{\frac{3j_3}{2j_4+2j_5}\frac{S[Cu^{2+}]_0}{j_1[O_2]}+1}\frac{d[AA]}{dt} = \frac{\frac{j_3}{2j_4+2j_5}\frac{S[Cu^{2+}]_0}{j_1[O_2]}+\frac{j_5}{2j_4+2j_5}}{\frac{3j_3}{2j_4+2j_5}\frac{S[Cu^{2+}]_0}{j_1[O_2]}+1}\left(\frac{V_{max}[Cu^{2+}]_0}{K_m+[Cu^{2+}]_0} + S[Cu^{2+}]_0\right) \quad (S30)$$

Eq S30 could be simply expressed as:

$$\left.\frac{d[\cdot OH]}{dt}\right|_f = \frac{A[Cu^{2+}]_0+B}{3A[Cu^{2+}]_0+1}\left(\frac{V_{max}[Cu^{2+}]_0}{K_m+[Cu^{2+}]_0} + S[Cu^{2+}]_0\right) = \frac{A[Cu^{2+}]_0+B}{3A[Cu^{2+}]_0+1}\frac{d[AA]}{dt} = \frac{A[Cu^{2+}]_0+B}{3A[Cu^{2+}]_0+1}[AA]_0 OP_{AA} \quad (S31)$$

Where parameters A and derived from multiple constants:

$$A = \frac{j_3}{2j_4 + 2j_5} \frac{S}{j_1[\mathrm{O_2}]}$$

$$B = \frac{j_5}{2j_4 + 2j_5}$$

Text S3. Differential equation solution for Fe induced ·OH formation process

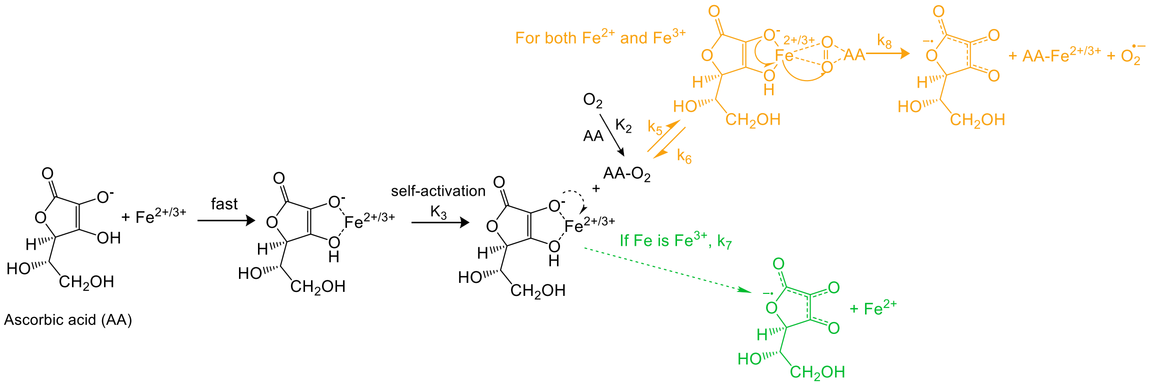

Scheme 2. Quasi-Michaelis–Menten mechanism for Fe-induced AA oxidation

For Fe cases, we need to include Fenton reaction:

$$H_2O_2 + Fe^{2+} \xrightarrow{j_3} \cdot OH + OH^- + Fe^{3+}$$

$$H_2O_2 + Fe^{3+} \xrightarrow{j_6} HO_2\cdot + H^+ + Fe^{2+}$$

Other reactions we considered for Fe induced ·OH radical production are the same as those in Cu induced ·OH generation:

$$Fe^{2+} + O_2 \xrightarrow{j_1} Fe^{3+} + \cdot O_2^-$$

$$\cdot O_2^- + H^+ \xrightarrow{j_2} \frac{1}{2}(H_2O_2 + O_2)$$

$$H_2O_2 \xrightarrow{j_4} H_2O + \frac{1}{2}O_2$$

$$H_2O_2 \xrightarrow{j_5} \cdot OH$$

Notice that these reactions are applicable for any Fe^{2+}/Fe^{3+} mixing ratio. Thus, we could express the differential equation about $\cdot O_2^-$ in a more general way:

$$\frac{d[\cdot O_2^-]}{dt} = P(\cdot O_2^-) + j_1[O_2][Fe^{2+}] - j_2[H^+][\cdot O_2^-] \quad (17)$$

Where $P(\cdot O_2^-)$ is the production rate of $\cdot O_2^-$ during Fe induced AA oxidation. For Fe and H_2O_2, the differential equations could be written as follows:

$$\frac{d[Fe^{2+}]}{dt} = -\frac{d[Fe^{3+}]}{dt} = S[Fe^{3+}] - j_1[O_2][Fe^{2+}] - j_3[H_2O_2][Fe^{2+}] + j_6[H_2O_2][Fe^{3+}] \quad (18)$$

$$\frac{d[H_2O_2]}{dt} = \frac{1}{2}j_2[H^+][\cdot O_2^-] - j_3[H_2O_2][Fe^{2+}] - j_4[H_2O_2] - j_5[H_2O_2] - j_6[H_2O_2][Fe^{3+}] \quad (19)$$

Linearly combining the two differential equations by eq 17 + eq 18 + 2*eq 19:

$$\frac{d[\cdot O_2^-]}{dt} + \frac{d[Fe^{2+}]}{dt} + 2\frac{d[H_2O_2]}{dt} = P(\cdot O_2^-) + S[Fe^{3+}] - [H_2O_2](3j_3[Fe^{2+}] + 2j_4 + 2j_5 + j_6[Fe^{3+}]) \quad (S32)$$

Applying PSSA for $\cdot O_2^-$, Fe, and H_2O_2 ($\frac{d[\cdot O_2^-]}{dt} = \frac{d[Fe^{2+}]}{dt} = \frac{d[H_2O_2]}{dt} = 0$):

$$[H_2O_2](3j_3[Fe^{2+}] + 2j_4 + 2j_5 + j_6[Fe^{3+}]) = P(\cdot O_2^-) + S[Fe^{3+}] \quad (S33)$$

The $\cdot OH$ formation rate we care about is $j_3[H_2O_2][Fe^{2+}] + j_5[H_2O_2]$:

$$\left.\frac{d[\cdot OH]}{dt}\right|_f = \frac{\frac{j_3}{2j_4+2j_5}[Fe^{2+}]+\frac{j_5}{2j_4+2j_5}}{\frac{3j_3}{2j_4+2j_5}[Fe^{2+}]+\frac{j_6}{2j_4+2j_5}[Fe^{3+}]+1}(P(\cdot O_2^-) + S[Fe^{3+}]) \quad (S34)$$

We could simply express eq S34 into:

$$\left.\frac{d[\cdot OH]}{dt}\right|_f = \frac{A[Fe^{2+}]+B}{3A[Fe^{2+}]+C[Fe^{3+}]+1}(P(\cdot O_2^-) + S[Fe^{3+}]) \quad (S35)$$

Where parameter A, B, and C are:

$$A = \frac{j_3}{2j_4 + 2j_5}$$

$$B = \frac{j_5}{2j_4 + 2j_5}$$

$$C = \frac{j_6}{2j_4 + 2j_5}$$

For Fe^{2+} samples, from Fig. 3a, we know that Fe^{3+} has a much lower AA depletion efficiency than Fe^{2+}, but we could not observe obvious reaction rate decrease over time in Fig. S2a. In addition, the measured cumulative $\cdot OH$ radical formation during the reaction is much smaller than the initial Fe^{2+} concentration. These suggest that Fe^{3+} concentration is negligible in our tested lab-prepared Fe^{2+} samples during the reaction process. Hence, we could ignore $C[Fe^{3+}]$ and $S[Fe^{3+}]$. Because of the same reason, $P(\cdot O_2^-)$ could be simply considered as a Michaelis–Menten like equation with respect to Fe^{2+} concentration. Based on these assumptions, we could simplify eq S34 as:

$$\left.\frac{d[\cdot OH]}{dt}\right|_f = \frac{A[Fe^{2+}]+B}{3A[Fe^{2+}]+1}\left(\frac{V_{max}[Fe^{2+}]}{K_m+[Fe^{2+}]}\right) \quad (S36)$$

For lab-prepared Fe^{3+} samples, we could transform $[Fe^{2+}]$ into $[Fe^{3+}]$ by eq 13:

$$[Fe^{2+}] = \frac{j_6[H_2O_2]+S}{j_1[O_2]+j_3[H_2O_2]}[Fe^{3+}] = \frac{j_6\frac{[H_2O_2]}{[O_2]}+\frac{S}{[O_2]}}{j_1+j_3\frac{[H_2O_2]}{[O_2]}}[Fe^{3+}] \approx \frac{S[Fe^{3+}]}{j_1[O_2]} \quad (S37)$$

With eq S37, we could rewrite eq S34 for Fe^{3+} samples:

$$\left.\frac{d[\cdot OH]}{dt}\right|_f = \frac{A'[Fe^{3+}]+B}{(3A'+C)[Fe^{3+}]+1}\left(\frac{V_{max}[Fe^{3+}]}{K_m+[Fe^{3+}]} + S[Fe^{3+}]\right) \quad (S38)$$

Where parameter A' is:

$$A' = \frac{S}{j_1[O_2]} A = \frac{j_3}{2j_4 + 2j_5} \frac{S}{j_1[O_2]}$$

References

(1) Wilson, R. J., Beezer, A. E., and Mitchell, J. C.: A kinetic study of the oxidation of L-ascorbic acid (vitamin C) in solution using an isothermal microcalorimeter, *Thermochim. Acta.* **1995**, 264, 27−40, https://doi.org/10.1016/0040-6031(95)02373-A.

(2) Yuan, X., Ninh Pham, A., Xing, G., L. Rose, A., and David Waite, T.: Effects of pH, Chloride, and Bicarbonate on Cu(I) Oxidation Kinetics at Circumneutral pH, *Environ. Sci. Technol.* **2012**, 46, 3, 1527–1535, https://doi.org/10.1021/es203394k.

Part B. Supporting figures and table

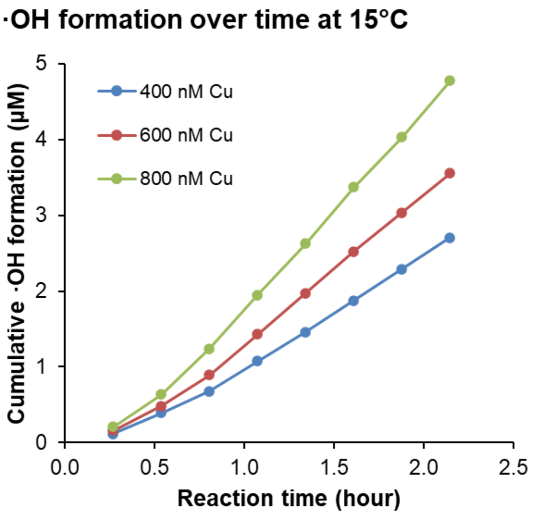

Fig. S1. A delayed zero-order kinetics with an equilibration period observed in ·OH generation process.

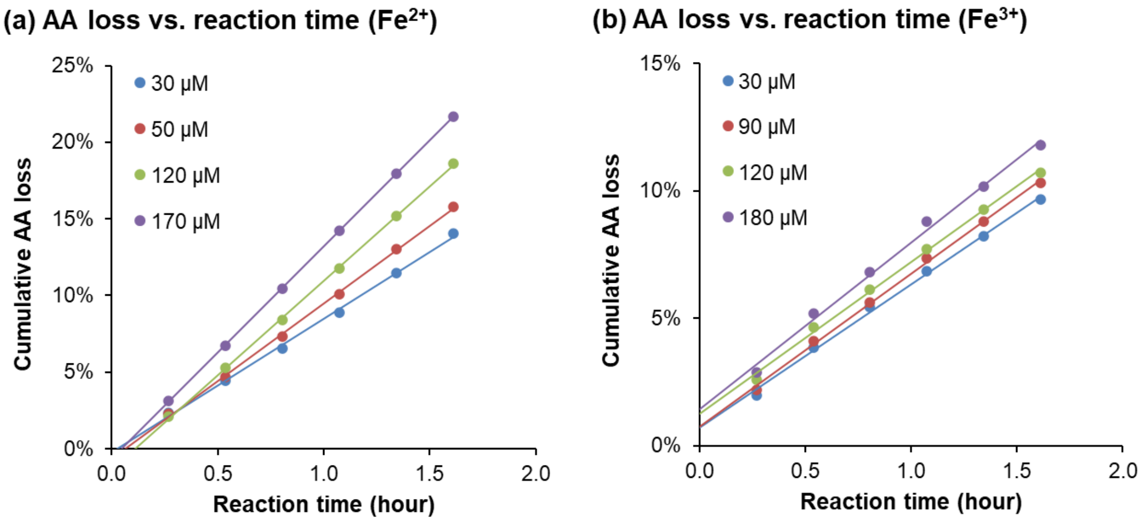

Fig. S2. Cumulative percentage AA loss curves in 1.6 hours: examples of (a) Fe^{2+} samples and (b) Fe^{3+} samples.

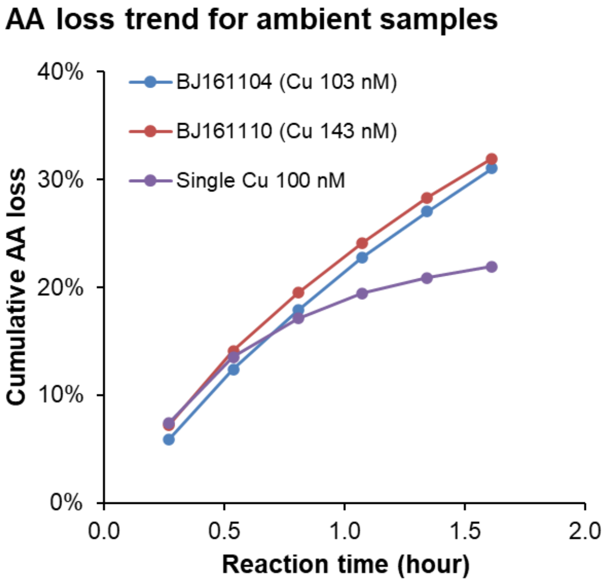

Fig. S3. Percentage AA loss trend comparison between ambient samples and single Cu solution with similar Cu level.

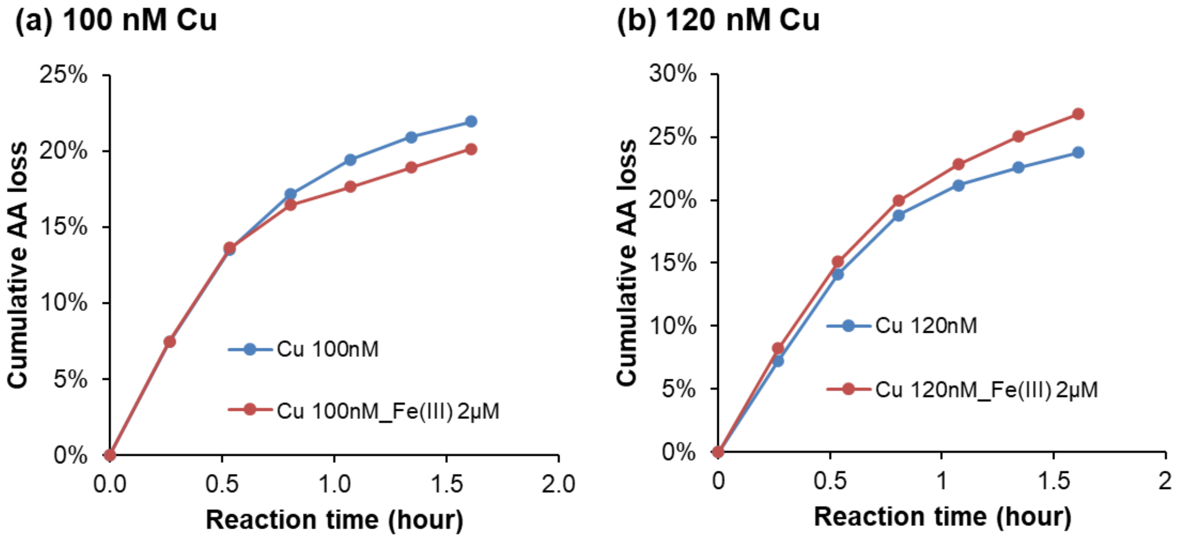

Fig. S4. Effect of Fe^{3+} addition on AA loss in Cu^{2+} solutions. (a) Comparison of percentage AA loss curves for 100 nM Cu^{2+} alone and 100 nM Cu^{2+} mixed with 2 µM Fe^{3+}. (b) Comparison of percentage AA loss curves for 120 nM Cu^{2+} alone and 120 nM Cu^{2+} mixed with 2 µM Fe^{3+}.In both cases, the addition of Fe^{3+} does not alter the AA loss curve relative to the single Cu^{2+} solution.

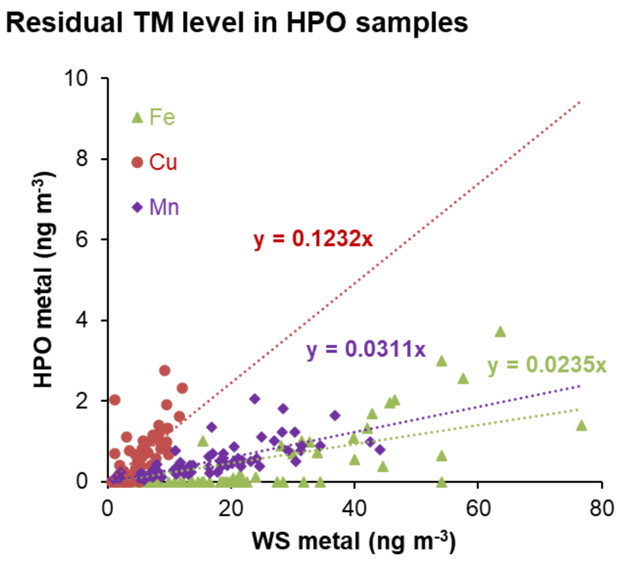

Fig. S5. Fe, Cu, and Mn concentration in HPO samples vs. WS samples

Table S1. Kinetic parameters calculated from our laboratory results

Parameters[a]	15°C Cu^{2+}	37°C Cu^{2+}	37°C Fe^{2+}	37°C Fe^{3+}	BJ 2016-17
K_m (nM)	56.77	0.25	$9.99*10^{4}$	$4.98*10^{3}$	-[b]
V_{max} (μM h^{-1})	35.03	5.40	42.32	3.64	-
S (h^{-1})	43.20	352.77	0	$9.39*10^{-3}$	186.66
A (nM^{-1})	$2.12*10^{-5}$	-	$2.68*10^{-7}$	$2.25*10^{-6}$	-
B (N.A.)	$2.22*10^{-2}$	-	$2.78*10^{-2}$	$3.21*10^{-2}$	-
C (nM^{-1})	0	-	0	$1.30*10^{-7}$	-

[a] Parameters in this column correspond to those in the equations below for calculating metal-induced OP_{AA} (μM h^{-1}) and $OP_{\cdot OH}$ (μM h^{-1}). Metal concentrations ([M]) are in the unit of nM:

$$OP_{AA}(\mu M\ h^{-1}) = \frac{V_{max}[M]}{K_m + [M]} + S[M]$$

$$OP_{\cdot OH}(\mu M\ h^{-1}) = \frac{A\,[M] + B}{(3A + C)\,[M] + 1}\left(\frac{V_{max}[M]}{K_m + [M]} + S[M]\right)$$

[b] Not applicable.
